# An alternative adaptation strategy of the CCA-adding enzyme to accept noncanonical tRNA substrates in *Ascaris suum*

**DOI:** 10.1016/j.jbc.2025.108414

**Published:** 2025-03-17

**Authors:** Valerie Thalhofer, Claudius Doktor, Lena Philipp, Heike Betat, Mario Mörl

**Affiliations:** Institute for Biochemistry, Leipzig University, Leipzig, Germany

**Keywords:** CCA-adding enzyme, tRNA nucleotidyltransferase, noncanonical tRNAs, miniaturized tRNAs, coevolution, convergent evolution

## Abstract

Playing a central role in translation, tRNAs act as an essential adapter linking the correct amino acid to the corresponding mRNA codon in translation. Due to this function, all tRNAs exhibit a typical secondary and tertiary structure to be recognized by the tRNA maturation enzymes as well as many components of the translation machinery. Yet, there is growing evidence for structurally deviating tRNAs in metazoan mitochondria, requiring a coevolution and adaptation of these enzymes to the unusual structures of their substrates. Here, it is shown that the CCA-adding enzyme of *Ascaris suum* carries such a specific adaptation in form of a C-terminal extension. The corresponding enzymes of other nematodes also carry such extensions, and many of them have an additional adaptation in a small region of their N-terminal catalytic core. Thus, the presented data indicate that these enzymes evolved two distinct strategies to tolerate noncanonical tRNAs as substrates for CCA incorporation. The identified C-terminal extension represents a surprising case of convergent evolution in tRNA substrate adaptation, as the nematode mitochondrial translation factor EF-Tu1 carries a similar extension that is essential for efficient binding to such structurally deviating tRNAs.

As universal adapter molecules, tRNAs help to translate the ORF of an mRNA into the amino acid sequence of the nascent protein, fulfilling a central function in protein synthesis in all kingdoms of life. For an efficient interaction of the tRNA pool with the large number of enzymes and proteins involved in tRNA maturation and translation, tRNAs exhibit highly conserved structural features (Fig. 1*A*). The cloverleaf-like secondary structure consists of an acceptor stem, the anticodon arm, as well as D- and T-arms. In the tertiary structure, tRNAs fold into an L-shaped form where acceptor stem and T-arm as well as D- and anticodon arms stack on each other, respectively (1, 2, 3). At the 3′-end, the invariant CCA triplet is found, representing the attachment site for the cognate amino acid to be delivered to the ribosome (4, 5).

**Figure 1:**
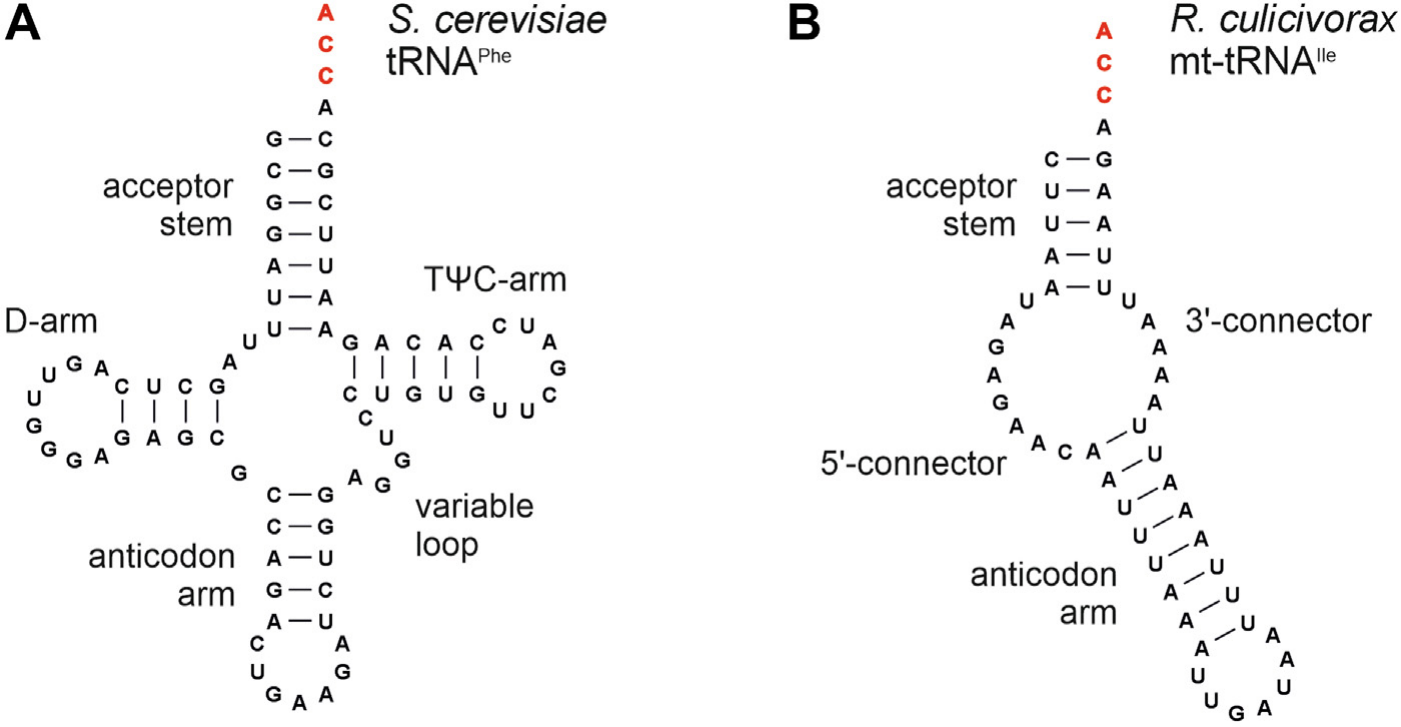
**Canonical and noncanonical tRNAs.***A*, tRNA^Phe^ from *Saccharomyces cerevisiae* represents a cloverleaf structure typical for canonical tRNA molecules and has a length of 76 nucleotides. The individual parts of this secondary structure are indicated. *B*, one of the most extremely truncated noncanonical tRNA is the mitochondrial tRNA^Ile^ from the nematode *Romanomermis culicivorax*. Acceptor and anticodon arms show an untypical length, D- and TψC-arms are replaced by unstructured connector elements, reducing the size of the tRNA down to 50 nucleotides. In both tRNAs, the post-transcriptionally added CCA-end is indicated in *red*.

Surprisingly, a number of metazoan mitochondrial tRNAs (mt-tRNAs) show considerable structural deviations, as they lack the D- or T-arm (6, 7, 8, 9). A prominent example is the mammalian mt-tRNA^Ser^(AGY), where the complete D-arm is missing (10). In the mitochondrial genome of nematodes, acari, and arachnids, the situation is even more extreme. Here, mt-tRNAs are described that lack either one or even both of these arms (8, 9, 11, 12, 13, 14, 15, 16). The best characterized armless mt-tRNAs are found in the enoplean nematode *Romanomermis culicivorax*, where functional tRNAs as short as 45 nts were identified (14, 17, 18). These transcripts exhibit a hairpin-like structure with a central bulge region consisting of single-stranded connector elements replacing the missing D- and T-arms (Fig. 1*B*). Interestingly, these noncanonical tRNAs fold into a three-dimensional boomerang-like shape, resembling the canonical L-shape described previously (18).

The presence of such structurally deviating tRNAs represents a particular challenge for the tRNA-interacting components of the translation machinery. While the individual mitochondrial aminoacyl-tRNA synthetases interact only with their cognate mt-tRNA, other proteins like translation factors or tRNA maturation enzymes must deal with the complete mt-tRNA pool. A special case is ATP(CTP):tRNA nucleotidyltransferase that adds the nonencoded CCA triplet to the 3′-end of all tRNAs (19, 20, 21). As the eukaryotic enzyme is encoded in a single nuclear gene, the corresponding protein represents both the cytosolic as well as the mitochondrial CCA-adding activity (22, 23, 24, 25). Hence, this enzyme must recognize two different tRNA pools—the cytosolic pool consisting of tRNAs with canonical structure as well as the mitochondrial one where also deviating tRNA structures exist.

Based on conserved sequence motifs, CCA-adding enzymes are members of the polymerase β superfamily and can be divided into class I (archaeal) and class II (bacterial and eukaryotic) enzymes (19, 20, 21, 26). These highly specific RNA polymerases have a unique mode of polymerization that is based on a single nucleotide-binding pocket without the involvement of a nucleic acid–based template (19, 27). In class II enzymes, the conserved catalytic core elements are located in the N-terminal region (27). The less conserved C-terminal part is involved in substrate binding and recognizes the elbow region of the tRNA L-shape, consisting of the D- and TΨC-loop (28, 29, 30). Consequently, armless mt-tRNAs lacking this region are not accepted by CCA-adding enzymes from organisms with exclusively canonically structured tRNAs (24).

It has been shown recently that the CCA-adding enzyme of *R. culicivorax*, where armless mt-tRNAs were identified, is specifically adapted to accept both canonical as well as miniaturized mt-tRNAs as substrates. In the catalytic core of this enzyme (*Rcu*CCA), a β-turn element, usually involved in primer 3′-end positioning, contributes to an enhanced tRNA binding, because of an enrichment in basic amino acids (24). As a result, armless tRNAs are readily accepted for CCA addition.

Another nematode with truncated mt-tRNAs is *Ascaris suum*. Twenty-one mt-tRNAs are lacking the T-arm, where one carries no D-arm (7, 9, 31). Hence, the CCA-adding enzyme *Asu*CCA is also expected to be adapted to noncanonical tRNA substrates. The enzyme carries a C-terminal extension with a noticeable enrichment in lysine and arginine residues (Fig. 2). Here, it was investigated whether this extended region represents an adaptation to truncated tRNAs and whether it even enables CCA incorporation into completely armless tRNA substrates. In a mutational analysis of the recombinant enzyme, the extension was identified as the main element involved in armless tRNA binding and CCA addition, whereas the β-turn element makes only a minor contribution to the recognition of such noncanonical tRNA substrates.

**Figure 2:**
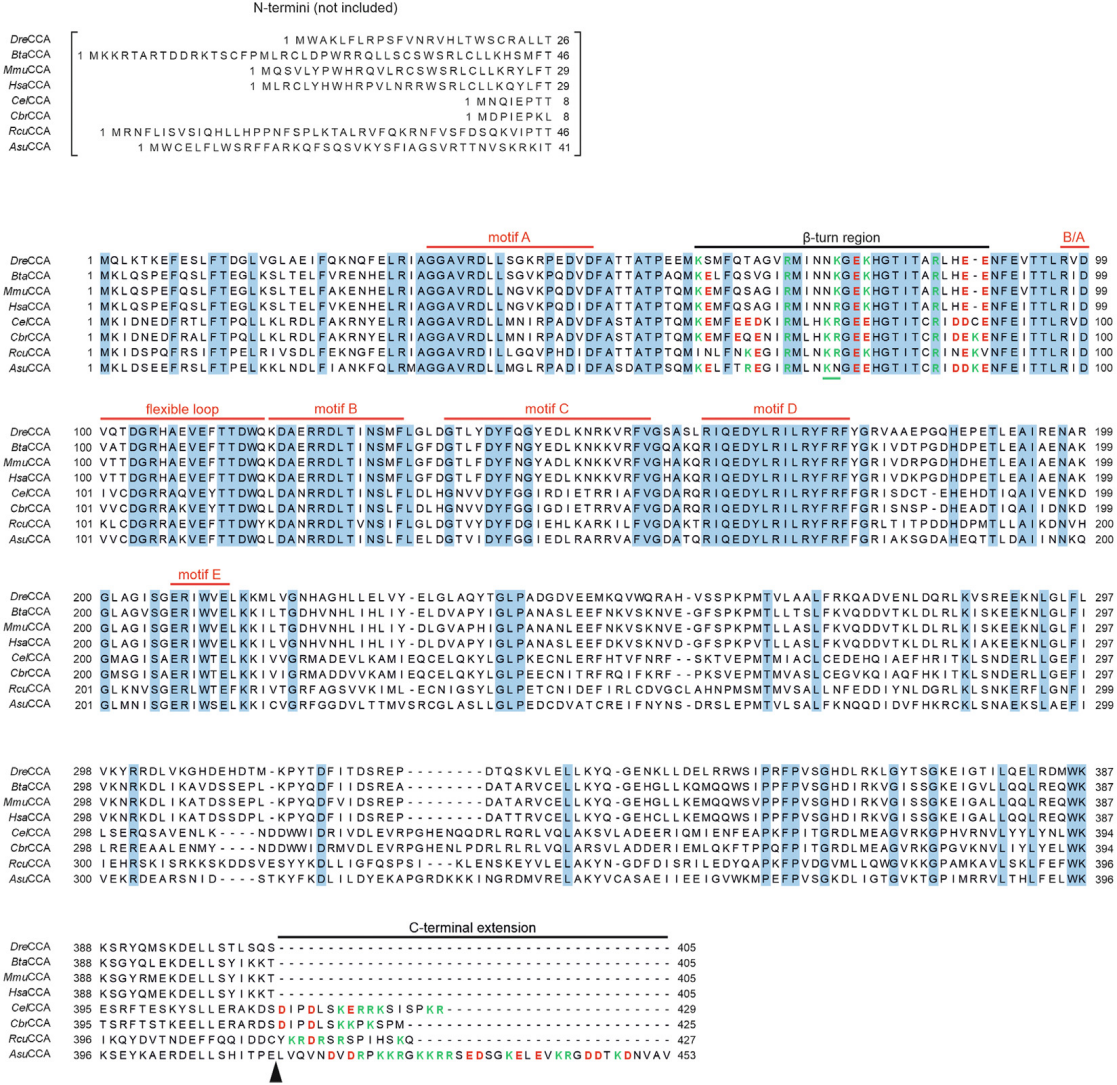
**Sequence alignment of CCA-adding enzymes from vertebrate and nematode representatives.** Enzyme sequences from *Danio rerio* (*Dre*CCA), *Bos taurus* (*Bta*CCA), *Mus musculus* (*Mmu*CCA), *Homo sapiens* (*Hsa*CCA), *Caenorhabditis elegans* (*Cel*CCA), *Caenorhabditis briggsae* (*Cbr*CCA), *Romanomermis culicivorax* (*Rcu*CCA), and *Ascaris suum* (*Asu*CCA) are shown. Identical residues are highlighted in *blue*, basic and acidic residues in the β-turn element and the C-terminal extension (*black bars*) are indicated in *green* and *red*, respectively. Catalytic core elements (*red bars*) are highly conserved in all shown CCA-adding enzyme sequences. Besides the five conserved motifs A to E, a flexible loop (involved in domain movement during CCA addition) and a basic/acidic motif (B/A, involved in proof reading of the incorporated nucleotides) are indicated. Only the nematode enzymes carry a C-terminal extension enriched in basic residues, with the longest one (40 positions) in *Asu*CCA. In the β-turn, *Asu*CCA is lacking one basic residue in the KR motif of the nematode sequences (*green bar*). The *black arrowhead* indicates the position for enzyme fusions and the C-terminal deletion variant. For a correct alignment, the variable N termini containing mitochondrial target sequences were omitted (number of positions indicated in *brackets*) (24).

Hence, the CCA-adding enzymes of nematodes with noncanonical truncated mt-tRNAs follow two different but efficient adaptational strategies to deal with two sets of structurally diverse tRNA substrates. To accept tRNAs that deviate from the canonical structure, an enrichment in positive charges is found either in a catalytic core element or in an extension of the tRNA-binding C terminus. Interestingly, while *A. suum* mt-tRNAs lack only one (D- or T-) arm, the *Asu*CCA enzyme accepts completely armless mt-tRNAs from *R. culicivorax* as substrates, indicating that the C-terminal extension likewise conveys a high affinity to these extremely truncated types of tRNA. This situation reminds of the coevolution of T-armless mt-tRNAs and mitochondrial elongation factor EF-Tu1 in nematodes, where a similar C-terminal extension is required for tRNA binding (32, 33, 34). Obviously, *Asu*CCA and nematode mt-EF-Tu1 proteins represent an interesting case of convergent evolution toward acceptance of such structurally deviating miniaturized tRNA substrates.

## Results

### *Asu*CCA carries a C-terminal extension essential for armless tRNA interaction

Noncanonical mt-tRNAs lacking D- or T-arm or even both arms are a hallmark of Nematoda (7, 9, 17). To accept such truncated tRNAs as substrates, the CCA-adding enzyme of the enoplean *R. culicivorax* (*Rcu*CCA) shows a specific adaptation in its catalytic core, where a β-turn carries several basic residues that confer efficient binding to armless tRNAs (24). To investigate whether this is a conserved feature in other nematode CCA-adding enzymes, a sequence alignment of several vertebrate and nematode enzymes was generated (Fig. 2). In the investigated proteins, the N-terminally located motifs A to E of the catalytic core are highly conserved, whereas the C-terminal part shows a much higher variation. In the β-turn, the nematode enzymes *Cel*CCA (*Caenorhabditis elegans*), *Cbr*CCA (*Caenorhabditis briggsae*), and *Rcu*CCA carry a motif of two basic residues KR. In contrast, *Asu*CCA carries the sequence KN with only one basic residue at the corresponding positions 74 and 75, a situation that is similar to that in the vertebrate enzymes, where an NK or NR motif is found.

A particularly striking difference in the worm sequences is the presence of a C-terminal extension, ranging from 13 (*Cbr*CCA) to 40 extra positions (*Asu*CCA). These extensions show an enrichment in positively charged basic residues, and in the *Ascaris* enzyme, most of these residues are concentrated in an elongated patch RPKKRGKKRR. A similar C-terminal extension is described for the mitochondrial elongation factor EF-Tu1 in several nematodes, where it is required for the interaction with tRNAs lacking the T-arm (33, 35). To investigate whether *Asu*CCA follows a similar strategy and uses this extension to recognize armless tRNAs for CCA addition, the functional impact of the extension was characterized in a comparative *in vitro* analysis with *Rcu*CCA, where the described β-turn is involved in tRNA binding. The human counterpart *Hsa*CCA served as a control, as this enzyme exclusively accepts canonical tRNAs and tRNAs lacking the D-arm for full CCA incorporation (24).

For these enzymes, codon-optimized ORFs were synthesized, recombinantly expressed in *Escherichia coli*, and purified. The enzymes lacked the N-terminal mitochondrial import signal, as this sequence can interfere with protein solubility, and its absence has no negative effect on the catalytic activity (23, 24, 36, 37). To investigate whether the removal of the C-terminal elongation has an effect on tRNA binding and CCA addition, the deletion variant *Asu*CCA ΔC40 (nomenclature according to Lusetti *et al.* (38)) was generated, representing residues 1 to 413 of the *A. suum* CCA-adding enzyme. To this end, a TAA stop codon was introduced at the corresponding position in the coding sequence of *Asu*CCA.

**Figure 3:**
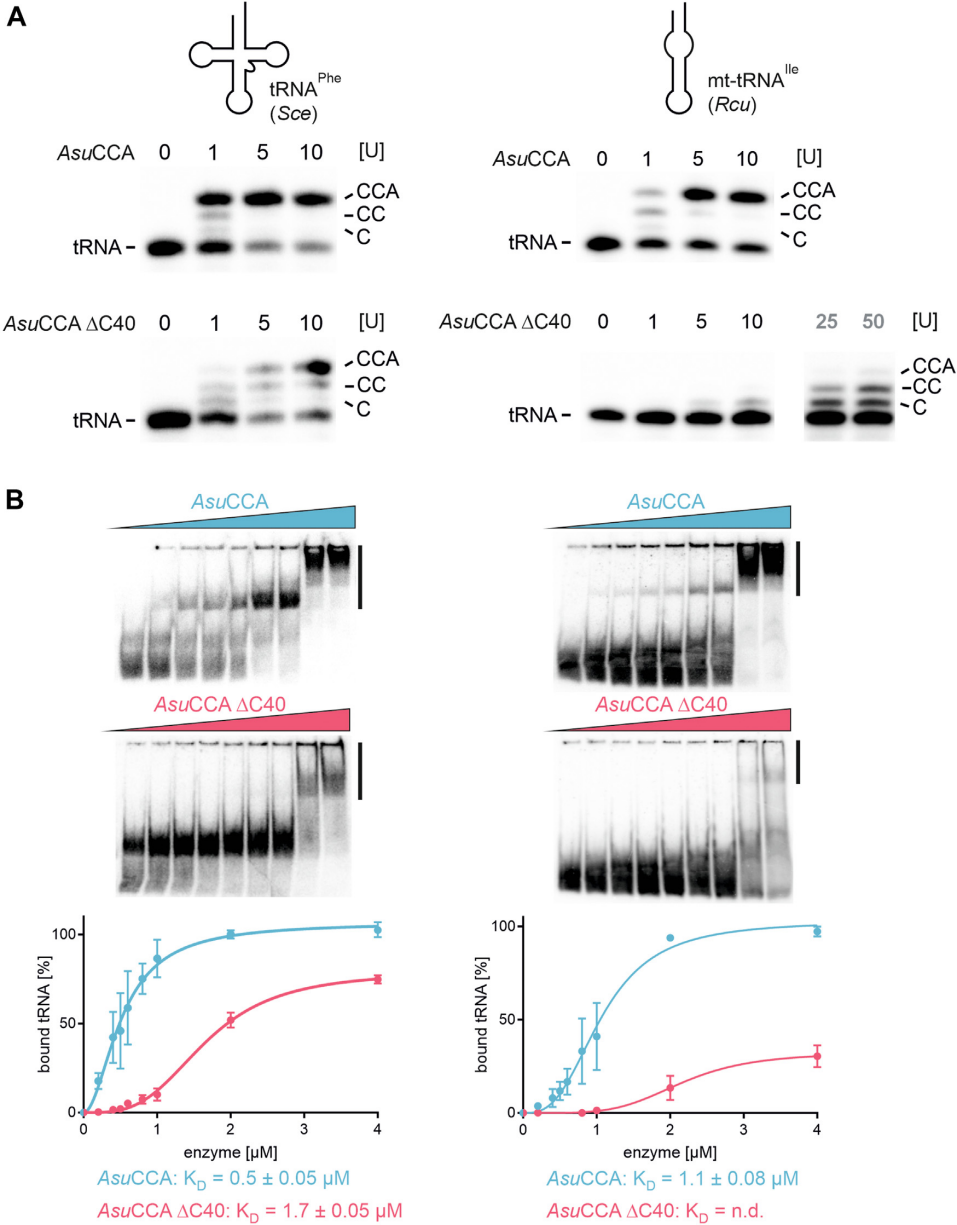
**Impact of the C-terminal extension on CCA-adding activity and tRNA substrate binding of *Asu*CCA.***A*, CCA addition on a canonical (*Sce* tRNA^Phe^ from *Saccharomyces cerevisiae*) and a noncanonical armless tRNA (tRNA^Ile^ from *Romanomermis culicivorax* mitochondria). The wt enzyme *Asu*CCA efficiently adds a complete CCA-end to both tRNA substrates at one or five arbitrary units, respectively (*upper gel panels*), whereas a version lacking the C-terminal 40 amino acids long extension (*Asu*CCA ΔC40) is only fully active on the canonical tRNA but adds just one to two C-residues on the armless tRNA, even at increased enzyme concentrations (25 and 50 U) (*lower panels*). On the noncanonical tRNA, a higher concentration of the wt enzyme is required for CCA addition, a fact that was also described for the corresponding enzyme of *R. culicivorax* (24). A quantitative comparison of these enzymatic activities and those of the following figures is shown in Fig. S1. *B*, quantitation of tRNA substrate interaction of *Asu*CCA wt (*blue*) and C-terminal deletion variant (*pink*). In the gel shift analysis, up to 4 μM of the recombinant protein were offered. Both enzyme versions bind efficiently to the canonical tRNA, resulting in dissociation constants of 0.5 to 1.7 μM (*left*), whereas only the wt enzyme shows high affinity for the armless substrate (*K*_*D*_ = 1.1 μM, *right*). In contrast, *Asu*CCA ΔC40 shows a dramatically reduced interaction with this tRNA that does not allow the determination of a dissociation constant (*right*). The observed supershifts at the highest concentration of *Asu*CCA are probably the result of protein dimer formation, a reaction that is frequently observed for CCA-adding enzymes at high concentrations *in vitro* (27, 36, 54). *K*_*D*_ values were determined by nonlinear regression and Hill slope fit. Data are means ± SD; n = 3.

Both *Asu*CCA and *Asu*CCA CΔ40 were tested *in vitro* for CCA-adding activity on two different tRNA substrates (Fig. 1). As a tRNA with canonical structure, yeast tRNA^Phe^ was used. This tRNA represents a well-established standard substrate, as the unmodified *in vitro* transcript folds into a structure almost identical to the corresponding *in vivo* tRNA (39). The second substrate was the hairpin-like mt-tRNA^Ile^ from *R. culicivorax*, where unstructured connector elements are found instead of D- and T-arm regions (18). With detailed biochemical and structural analyses, this transcript is one of the best characterized noncanonical tRNAs (14, 18), whereas almost all others are computational predictions based on mitochondrial genome sequences (7, 12, 16, 17, 31). Therefore, this transcript was chosen as a model substrate, although it is even more truncated than the mt-tRNAs of *A. suum*. Both ^32^P-labeled substrates lacking the CCA terminus were incubated in the presence of all four NTPs and 1 to 10 arbitrary units of the respective recombinant enzyme. Reaction products were separated on a denaturing polyacrylamide gel and visualized by autoradiography (Fig. 3*A*; for an easier comparison, all reaction products of these and the following enzymatic assays are quantified in Fig. S1). wt *Asu*CCA added a complete CCA-triplet to both the canonical tRNA^Phe^ and the armless mt-tRNA^Ile^. In contrast, *Asu*CCA ΔC40 catalyzed a complete CCA addition at moderate efficiency on tRNA^Phe^, whereas it was almost inactive on the armless mt-tRNA^Ile^. Even at higher enzyme concentrations of 25 and 50 arbitrary units, *Asu*CCA ΔC40 showed only a weak addition of two C-residues and almost no incorporation of the terminal A, indicating that the C-terminal extension is essential to accept the hairpin-like tRNA as a substrate.

To investigate whether the restricted activity of *Asu*CCA ΔC40 results from reduced substrate binding, EMSAs with wt *Asu*CCA and *Asu*CCA ΔC40 were performed, using both canonical and armless tRNA substrates. Radioactively labeled tRNA *in vitro* transcripts were incubated with increasing amounts of *Asu*CCA and *Asu*CCA ΔC40 (0–4 μM) and separated on a nondenaturing polyacrylamide gel (Fig. 3*B*). Quantitation of enzyme-bound and free tRNA substrates showed an efficient binding of *Asu*CCA to the canonical tRNA^Phe^ as well as to the armless mt-tRNA^Ile^, resulting in dissociation constants (*K*_*D*_) of 0.5 μM and 1.1 μM, respectively. *Asu*CCA ΔC40, in contrast, still recognizes the canonical tRNA^Phe^ with a *K*_*D*_ of 1.7 μM, while its affinity to the armless substrate is dramatically reduced, so that no dissociation constant could be determined. Taken together, these data demonstrate that the C-terminal extension is not required for catalysis but allows an improved substrate interaction and CCA addition on structurally deviating tRNAs.

### The C-terminal extension enables *Hsa*CCA to process armless tRNAs

**Figure 4:**
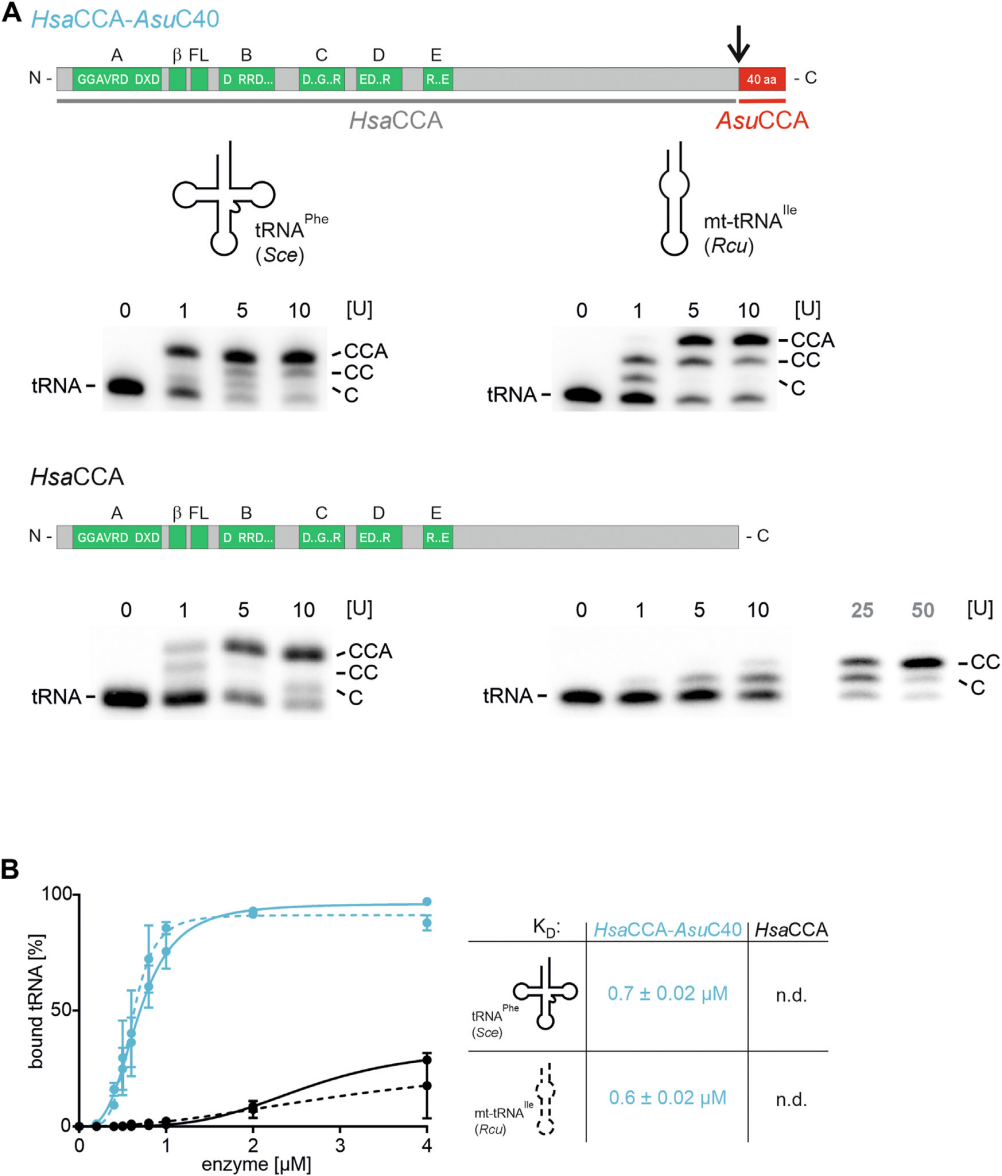
**The human CCA-adding enzyme carrying the C-terminal extension of *Asu*CCA accepts an armless tRNA as substrate.***A*, when fused to the C-terminal extension of *Asu*CCA, the human enzyme readily accepts canonical (*left*) as well as structurally deviating tRNA substrates (*right*) for efficient CCA-addition. In contrast, the human wt enzyme (*lower gel panels*) adds only two C-residues to the armless tRNA but not a complete CCA-end (*right*). On the canonical tRNA, it adds complete CCA-ends, although at a somewhat lower efficiency than the chimera (*left*). In the bar representation of the enzymes, catalytic core motifs are shown in *green*, the fusion position of the *Ascaris suum* C-terminal extension (*red*) is indicated by the *black arrow*. *B*, as described in the literature, the human wt enzyme has a very low affinity to tRNA substrates in general, so that no binding parameters can be determined (*black*). The C-terminal extension (*blue*), however, conveys a dramatically increased affinity to both canonical (*K*_*D*_ = 0.7 μM) as well as armless tRNA (*K*_*D*_ = 0.6 μM). Data for wt *Hsa*CCA are taken from Hennig and Philipp (24).

Since the C-terminal extension is a prerequisite for *Asu*CCA to add a CCA-end to the armless tRNA substrate, we investigated whether this element also confers this property to a CCA-adding enzyme that *per se* only accepts canonical tRNAs as substrates. To this end, the 40 amino acids long extension of *Asu*CCA was transplanted to the human CCA-adding enzyme *Hsa*CCA (Fig. 4*A*). The resulting chimera *Hsa*CCA-*Asu*C40 was recombinantly expressed and purified and subsequently tested on both the canonical tRNA^Phe^ and the mt-tRNA^Ile^ lacking the CCA terminus. The ^32^P-labeled substrates were incubated in the presence of NTPs and 1 to 10 arbitrary units of the recombinant enzyme. The reaction products were separated by PAGE and visualized by autoradiography. *Hsa*CCA was fully active on tRNA^Phe^ but added only one to two C residues to mt-tRNA^Ile^ even at elevated enzyme concentrations, as observed by Henning *et al.* (24). The addition of the C-terminal extension of *Asu*CCA, however, resulted in highly efficient CCA synthesis on both canonical as well as armless tRNA (Fig. 4*A*).

To investigate whether the CCA addition on the armless tRNA correlates with an efficient substrate binding because of the presence of the C-terminal extension in *Hsa*CCA-*Asu*C40, dissociation constants for both tRNA substrates were determined by gel shift analysis (Fig. 4*B*). As described in the literature, substrate binding of wt *Hsa*CCA is rather weak and does not allow for a *K*_*D*_ value determination (24). In contrast, *Hsa*CCA carrying the C-terminal extension exhibits a high substrate affinity, resulting in *K*_*D*_ values of 0.7 and 0.6 μM for the canonical and the armless tRNA, respectively.

### In *Asu*CCA, the **β**-turn in the catalytic core makes only a minimal contribution to armless tRNA recognition and CCA incorporation

**Figure 5:**
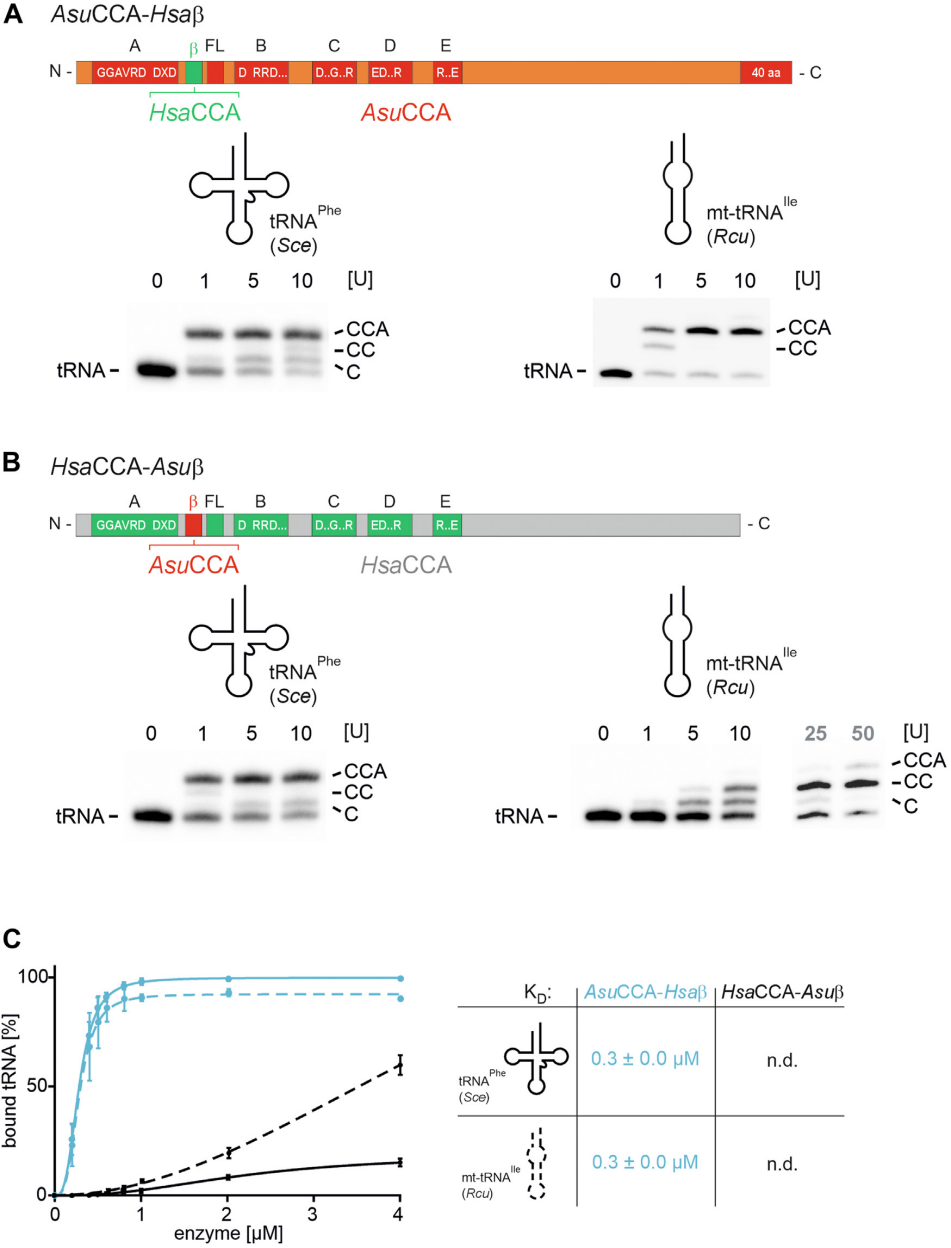
**The β-turn of *Asu*CCA does not convey an efficient CCA addition to a noncanonical tRNA.***A*, when the β-turn of *Asu*CCA (*orange/red*) is replaced by the corresponding element of *Hsa*CCA (*green*), the resulting chimera *Asu*CCA-*Hsa*β exhibits an unaltered activity on both canonical (*left*) and noncanonical tRNA substrate (*right*), leading to complete CCA addition with only one arbitrary enzyme unit. This result shows that the extension (*red*), which is still present in the chimera, is essential for the recognition of the structurally deviating tRNA. *B*, in the reciprocal chimera *Hsa*CCA-*Asu*β, the *Asu*CCA β-turn has almost no effect on the activity of *Hsa*CCA. The enzyme is fully active on the cloverleaf tRNA (1 arbitrary unit is sufficient for complete CCA synthesis, *left*). On the armless substrate, the chimera adds two C-residues, and only at higher enzyme concentrations, a very weak A-incorporation is observed (*right*). *C*, reciprocal β-turn replacements do not affect substrate-binding behavior. *K*_*D*_ values could only be determined for *Asu*CCA-*Hsa*β (*cyan*), as this chimera still carries the C-terminal extension that conveys efficient tRNA binding.

Previous experiments have shown that the CCA-adding enzyme from the closely related nematode *R. culicivorax* uses a small β-turn in its catalytic core to recognize and bind armless tRNAs. This element was originally identified to position the priming tRNA 3′-end for nucleotide incorporation (40). When transplanted into the human enzyme, this element confers a significantly improved tRNA affinity, resulting in full CCA addition on armless tRNAs (24). To investigate whether the corresponding region plays a similar role in *Asu*CCA substrate recognition and catalysis, we reciprocally exchanged this element in *Asu*CCA and *Hsa*CCA, resulting in chimeras *Asu*CCA-*Hsa*β (*Asu*CCA carrying the β-turn of *Hsa*CCA) and *Hsa*CCA-*Asu*β (*Hsa*CCA carrying the β-turn of *Asu*CCA) (Fig. 5). Surprisingly, these replacements had almost no effect on the catalytic activity of the β-turn chimeras. *Asu*CCA-*Hsa*β accepted both the canonical as well as the armless tRNA substrate for full CCA addition (Fig. 5*A*), whereas 10 units of the human enzyme carrying the β-turn of *Asu*CCA still added only two C-residues to the armless tRNA (Fig. 5*B*). Only at 25 and 50 units, a faint additional band appeared, representing a complete but highly ineffective CCA addition. Hence, the β-turn of *Asu*CCA has only a very minor effect on substrate specificity. Furthermore, the β-turn replacements also had no effect on tRNA binding (Fig. 5*C*). For *Asu*CCA-*Hsa*β, a dissociation constant of 0.3 μM was determined for both canonical and armless tRNA, whereas for *Hsa*CCA-*Asu*β, tRNA interaction was too weak to identify binding constants, resembling the binding behavior of wt *Hsa*CCA.

This result indicates that the two nematode enzymes, *Asu*CCA and *Rcu*CCA, follow different evolutionary strategies to accept noncanonical tRNAs for CCA addition. *Asu*CCA uses the C-terminal extension to recognize such tRNAs as substrates, wheras its β-turn hardly contributes to this interaction. In *Rcu*CCA, however, the β-turn is the essential element for CCA addition to these transcripts. Yet, the sequence alignment (Fig. 2) indicates that also *Rcu*CCA carries a C-terminal extension, although this region is shorter than in *Asu*CCA and consists of only 14 amino acid residues, including two lysine and three arginine positions.

### The C-terminal extension of *Rcu*CCA and its impact on CCA addition

**Figure 6:**
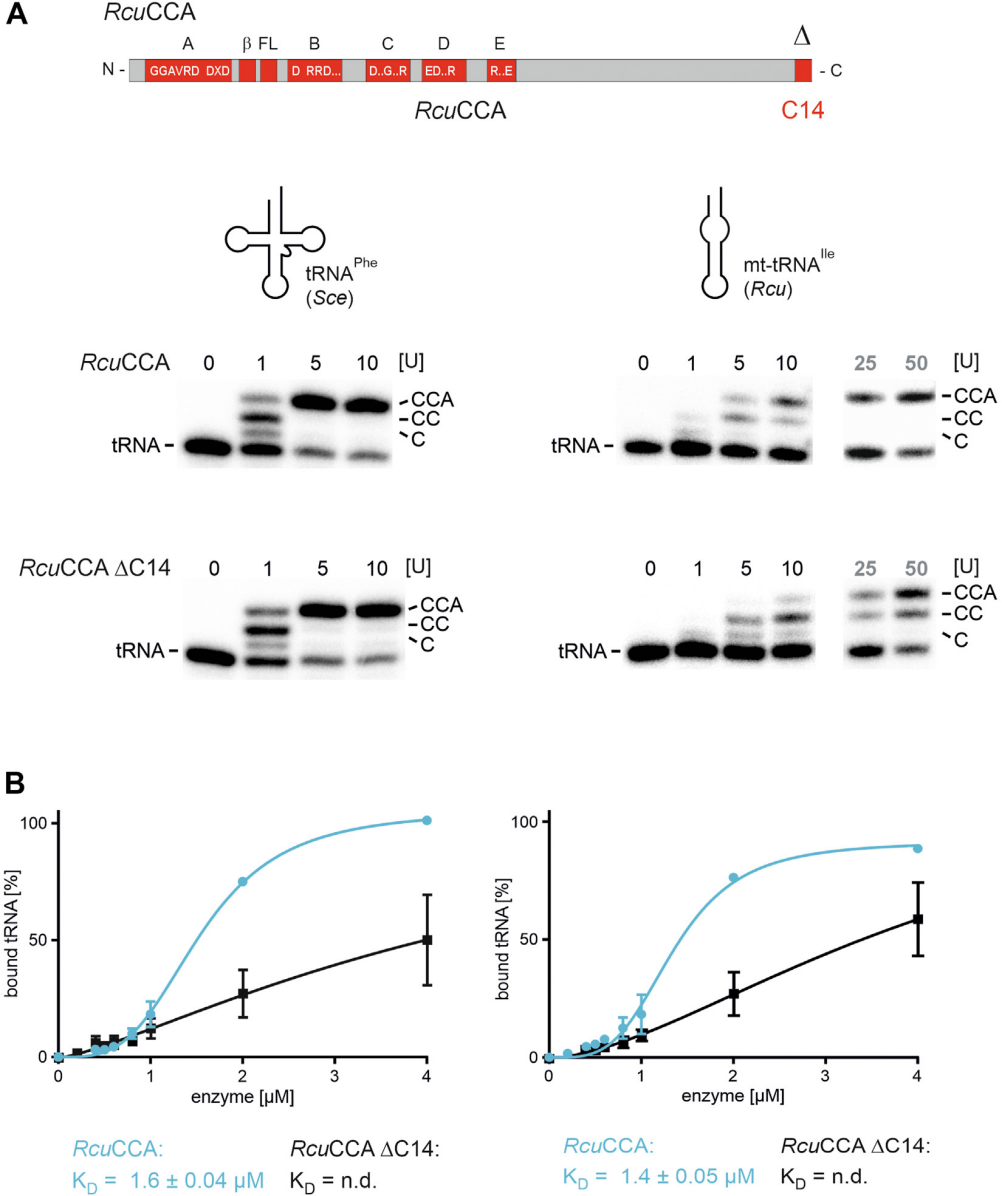
**In *Rcu*CCA, the short C-terminal extension makes only a minor contribution to CCA addition to a noncanonical tRNA.***A*, on the canonical tRNA^Phe^, *Rcu*CCA lacking the C-terminal extension of 14 amino acids (*Rcu*CCA ΔC14) is similarly active as the wt enzyme (*Rcu*CCA). On the armless mt-tRNA^Ile^, the activity of this deletion variant is somewhat reduced but still leads to complete CCA addition, especially at elevated enzyme concentrations of 25 and 50 U. *B*, in contrast to the polymerization activity, substrate binding is strongly reduced in *Rcu*CCA ΔC14 (*black curves*), so that no dissociation constants could be determined. The wt enzyme (*blue curves*), however, binds both canonical as well as armless tRNA substrates at a *K*_*D*_ of 1.6 and 1.4 μM, respectively. mt-tRNA, mitochondrial tRNA.

**Figure 7:**
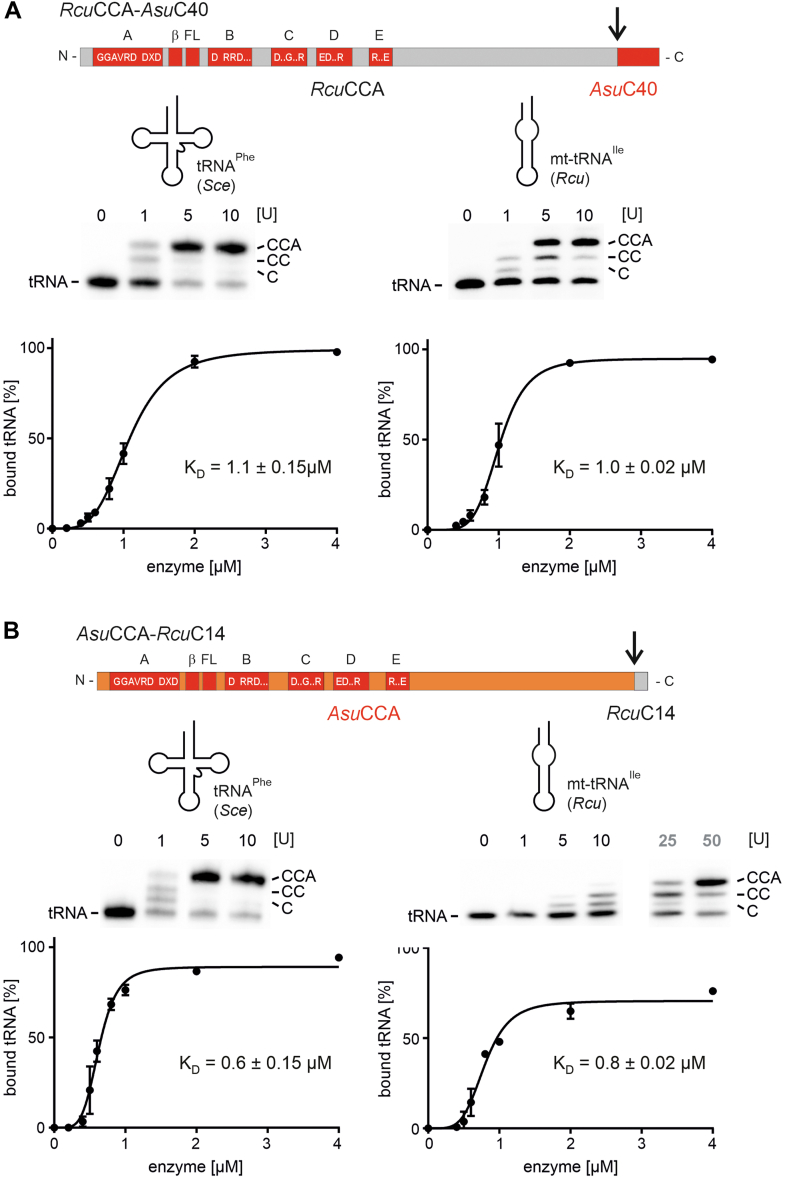
**Reciprocal exchange of C-terminal extensions between *Asu*CCA and *Rcu*CCA.***A*, when introduced into *Rcu*CCA, the C40 extension of *Asu*CCA does not affect catalysis or substrate interaction, supporting the observation that in this enzyme the β-turn element is the major adaptation to noncanonical tRNA substrates. *B*, for *Asu*CCA, replacing the C40 extension with the C14 region of *Rcu*CCA has no effect on CCA addition to the standard tRNA substrate, whereas CCA incorporation to the armless tRNA is reduced and only visible at high enzyme concentrations (25 and 50 U). However, the affinity of the enzyme chimera to both substrates is not affected. This result suggests that the C14 region of *Rcu*CCA is involved in efficient substrate binding but makes a smaller contribution to CCA addition on the armless tRNA compared with the C40 region of *Asu*CCA.

While the N-terminally located β-turn of *Rcu*CCA represents the major adaptation to armless tRNA substrates, previous results suggest that the C-terminal half of the enzyme also contributes to CCA addition, albeit only to a minor extent (24). To address this contribution in more detail, we deleted the C-terminal 14 residue extension and tested the resulting variant *Rcu*CCA ΔC14 for activity (Fig. 6*A*). On the canonical tRNA, this enzyme version exhibited wt-like activity. On the armless tRNA, nucleotide incorporation was reduced but still led to complete CCA addition, especially at elevated enzyme concentrations. Again, these findings corroborate the results of Henning *et al.* (24). However, binding to both types of tRNA was considerably affected, so that no dissociation constants could be determined (Fig. 6*B*). This indicates that in *Rcu*CCA, the C-terminal extension—in addition to the β-turn—contributes to substrate binding but is less important for a complete CCA synthesis. In contrast, the extension of *Asu*CCA is essential for armless tRNA binding as well as full CCA addition (Fig. 3). To confirm this, the C-terminal extensions of *Asu*CCA and *Rcu*CCA were reciprocally exchanged, resulting in enzyme variants *Asu*CCA-*Rcu*C14 and *Rcu*CCA-*Asu*C40, respectively. The enzymes showed efficient binding to both tRNA substrates, with *K*_*D*_ values between 0.6 and 1.1 μM (Fig. 7). Furthermore, *Rcu*CCA-*Asu*C40 showed full CCA-adding activity on the cloverleaf and the armless tRNA (Fig. 7*A*). Here, both the adapted β-turn of *Rcu*CCA and the C40 extension of *Asu*CCA contribute to binding and 3′-end positioning of the tRNA. In contrast, *Asu*CCA-*Rcu*C14 was less efficient in CCA addition on the armless tRNA. However, at 25 and 50 units, it synthesized a complete CCA-end, indicating that the C14 extension supports substrate binding as well as CCA addition (Fig. 7*B*). The activity of *Asu*CCA-*Rcu*C14 shows that the β-turn of *Asu*CCA is not well adapted to armless tRNAs, whereas the C14 extension of *Rcu*CCA exhibits a certain contribution to these substrates, although not as efficient as the C40 extension of *Asu*CCA.

## Discussion

### The C-terminal extension of *Asu*CCA is an evolutionary adaptation to process noncanonical tRNA substrates

Representing the site of aminoacylation, the CCA-end is an essential element in all tRNA molecules (41). Consequently, the single CCA-adding enzyme responsible for its synthesis and maintenance must recognize the tRNA pools in the cytosol, mitochondria, as well as in chloroplasts. Cytosolic and plastid tRNAs fold into the cloverleaf secondary and L-shaped tertiary structure and represent therefore a readily recognizable consensus shape (2). In metazoan mitochondria, however, tRNAs can strongly deviate from the consensus, lacking D-, T-, or even both arms (1, 9, 13, 14, 15, 42). Such noncanonical structures represent a challenge for the CCA-adding enzyme. In nematodes, where deviating tRNA shapes are frequently found in mitochondria (16), the enzyme underwent an evolutionary adaptation to recognize these aberrant tRNA structures. In *R. culicivorax*, a β-turn element in the catalytic core represents such an example. The original function of this element is to position the tRNA 3′-end as a primer for correct A-addition during CCA synthesis (40). In *Rcu*CCA, the element carries two consecutive basic amino acids K74R75 that increase the binding of hairpin-like tRNAs, resulting in robust and efficient CCA synthesis (24). While we observe a similar composition of this loop in other nematode CCA-adding enzymes *Cel*CCA and *Cbr*CCA, the corresponding enzyme of *A. suum* carries only a single lysine residue K74, representing a situation that is found in CCA-adding enzymes dealing only with canonical tRNA structures (Fig. 2). Accordingly, this β-turn does not confer full CCA-adding activity on armless tRNAs when transplanted to the human enzyme and is probably primarily required in its original function of primer positioning (Fig. 5). However, when the K74R75 motif of the *Rcu*CCA β-turn was introduced into *Asu*CCA lacking the C-terminal extension (*Asu*CCA ΔC40), the resulting variant showed increased CCA-adding activity on armless tRNA (Fig. S2). In addition, the KR pair also improved substrate binding, although to a lesser extent. This result confirms that the β-turn of *Rcu*CCA is the main adaptation to noncanonical tRNA substrates.

The alignment in Figure 2 shows that *Asu*CCA carries a C-terminal extension of 40 residues, whereas the other enzymes either carry no extension (non-nematode enzymes) or a rather short extra region (enzymes from *C. elegans*, *Caenorhabditis britovi*, and *R. culicivorax* and other nematodes, Fig. S4). This extension has a dramatic impact on *Asu*CCA and results in highly efficient substrate binding and CCA addition on armless tRNAs (Fig. 3). Hence, the *A. suum* enzyme follows an adaptation strategy to noncanonical tRNA substrates that differs from that of *Rcu*CCA. Instead of increasing the basic character of the β-turn to enhance electrostatic attraction between negatively charged tRNA and the positively charged enzyme region, the extended C terminus is responsible for such an enhanced tRNA interaction. Again, an enrichment of basic residues seems to be involved in this binding. As the CCA-adding enzyme as well as the tRNA carry out intense domain movements during the switch from C- to A-addition (28, 43, 44, 45), it is conceivable that a structurally deviating tRNA is not bound tightly enough by an enzyme specific for canonical L-shaped tRNAs (24). As a result, such an enzyme adds only one or two C-residues to the armless tRNA but seems to lose contact to this substrate when the rearrangement for A-incorporation is induced. In *Asu*CCA and *Rcu*CCA, the C-terminal extension (*Asu*CCA) and the β-turn (*Rcu*CCA) efficiently compensate for this so that the tRNA remains bound during the whole CCA polymerization process.

The strategy of a C-terminal extension carrying basic residues is also found in other enzymes interacting with noncanonical mt-tRNAs. Like the CCA-adding enzyme, the mitochondrial translation elongation factor mt EF-Tu recognizes the complete tRNA pool. It binds to aminoacylated tRNAs and delivers them to the ribosome. In nematodes, only the D-armless tRNA for serine requires a specific mt EF-Tu2 protein for its participation in mitochondrial protein synthesis (46). The residual 19 tRNAs are recognized by mt EF-Tu1. To interact with the varying structures in these mt-tRNAs (cloverleaf shape, T-armless, and completely armless hairpin-like tRNAs), mt EF-Tu1 carries an extension of 41 (*Trichinella britovi*) to 57 (*C. elegans*) extra amino acid positions at the C terminus (32, 34, 35, 47) (Fig. S4*A*). An enrichment of basic residues similar to that in *Asu*CCA seems to be responsible for binding to the noncanonical tRNAs, compensating for the loss of the T-arm region. However, while the mt EF-Tu1 extensions are highly conserved and probably have a common evolutionary origin, this is not the case for the extensions of CCA-adding enzymes (Fig. S4*B*). Besides the fact that they carry a high proportion of basic residues, they show neither sequence similarities nor a comparable length. Thus, although both extensions appear to fulfill a similar function, they are not evolutionary related, and detailed structure analyses are required to identify their mode of function and tRNA interaction.

**Figure 8:**
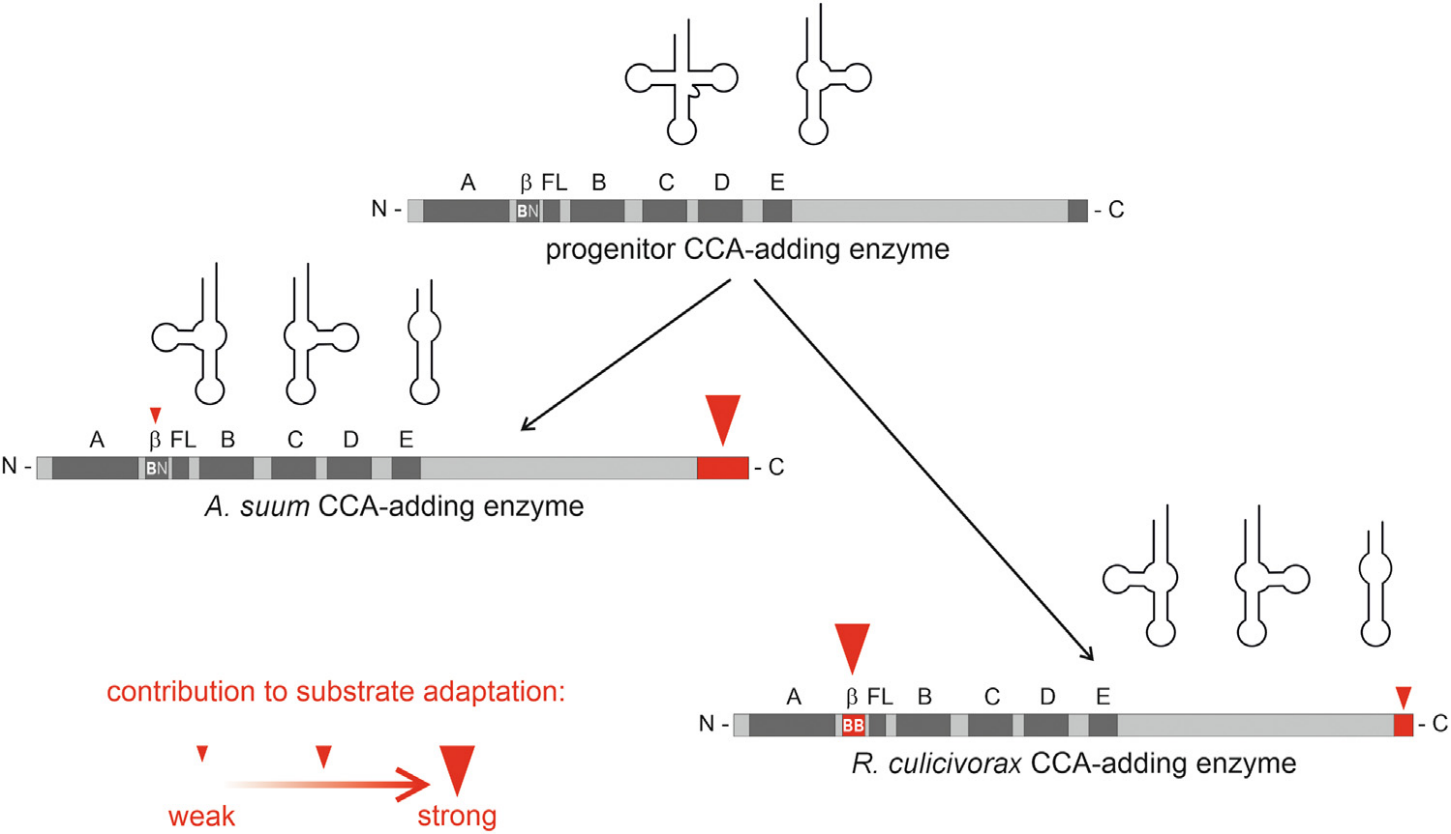
**Evolutionary scenario: The CCA-adding enzymes of nematodes follow two different adaptation strategies to accept noncanonical tRNA substrates.** Since truncated tRNAs are an acquired trait (16), the original CCA-adding enzyme probably recognized only canonical and the widely distributed unique D-armless tRNA^Ser^. In the CCA-adding enzyme of *Ascaris suum*, a C-terminal extension evolved as a prerequisite for the acceptance of other structurally deviating tRNAs lacking D-, T-, or even both arms (*large red arrowhead*). In contrast, the β-turn showed almost no adaptation as it carries only a single basic residue (BN; B = any basic residue; N = any amino acid except basic arginine or lysine; *small red arrowhead*). This element therefore has its original function in positioning the 3′-end of the tRNA in the catalytic core. In the corresponding enzyme of *Romanomermis culicivorax*, the main adaptation to noncanonical tRNAs is represented by the two basic positions in the β-turn (BB, *large red arrowhead*). The small C-terminal extension in this enzyme contributes to the adaptation but to a lesser extent (*intermediate red arrowhead*).

Interestingly, in *Rcu*CCA, the β-turn as well as the C-terminal extension strategy are combined, although the latter contributes to a lesser extent to armless tRNA acceptance. This is documented by the fact that CCA addition by *Asu*CCA is less efficient when the enzyme carries the 14-residue extension of *Rcu*CCA instead of its original 40 residues (Fig. 7). Instead, *Asu*CCA relies primarily on its long extension and has no obvious adaptation in its β-turn region, as this part hardly conveys full CCA addition to armless tRNAs when inserted into the human enzyme (Fig. 5*B*). Thus, the current data suggest that CCA-adding enzymes can utilize two regions, the β-turn and the C-terminal extension, for adaptation to structurally deviating tRNA substrates. Due to the positively charged residues in these elements, electrostatic attraction to the tRNA enables a tight substrate binding to ensure the production of functional tRNAs with a CCA-end. As such truncated tRNA transcripts exist only in nematode and arachnids (16), they obviously represent a derived state, and their cloverleaf-structured counterparts are considered as ancestral. Accordingly, the ancestral CCA-adding enzyme only dealt with the canonical tRNA shape, and the nematode enzymes co-volved with the occurrence of noncanonical tRNA structures. This is supported by the fact that a reconstructed ancestral class II CCA-adding enzyme does not accept hairpin tRNAs for CCA addition (48). Starting from such a progenitor CCA-adding enzyme, *Asu*CCA obtained the C-terminal extension to accept the deviating mt-tRNA substrates, whereas the corresponding enzyme in *R. culicivorax* mainly evolved a specific β-turn sequence whose substrate binding is supported by a small extension (Fig. 8).

Taken together, the nematode CCA-adding enzymes follow two distinct evolutionary pathways in adapting to their unusually shaped tRNA substrates. Sequence analysis of 38 CCA-adding enzymes from all available nematode clades shows that a C-terminal extension rich in basic amino acid residues is a common feature, whereas the pair of basic residues located in the β-turn element is not always found (Fig. S3). Neither length nor the percentage of basic positions of the C-terminal extension allow an estimation of an individual contribution or a concerted evolution of these two elements to substrate adaptation. Here, detailed additional biochemical data of a much greater number of enzymes are required.

Interestingly, although *A. suum* mitochondria only carry D- or T-arm lacking tRNAs, *Asu*CCA readily accepts completely armless hairpin-structured transcripts as well. This indicates that the mt-tRNAs might further evolve into armless tRNAs without affecting the viability of *A. suum*. Structural analyses are now required to fully understand the different tRNA-binding modes of these enzymes, and it will be interesting to see whether other CCA-adding enzymes in nematodes, spiders, and mites have evolved further strategies to accept these noncanonical substrates.

## Experimental procedures

### Sequence alignments

Protein sequences of CCA-adding enzymes were identified using the BLAST tool at the National Center for Biotechnology Information website (https://blast.ncbi.nlm.nih.gov/Blast.cgi). The *Rcu*CCA sequence was used as described (24). The retrieved sequences were aligned using the web tool Clustal Omega with default parameter settings (49). The alignment was visualized with JalView (50).

### Construction of recombinant enzymes

ORFs of CCA-adding enzymes from *A. suum*, *R. culicivorax*, and *Homo sapiens* were codon-optimized for expression in *E. coli* and synthesized by GenScript. ORFs lacking the N termini containing the mitochondrial target signals were inserted into pET28a(+), resulting in constructs with an N-terminal His_6_ tag. For generation of the deletion variant, a stop codon (TAA) was introduced into the coding sequence of the *Asu*CCA enzyme using the QuikChange Site-Directed Mutagenesis Kit (Agilent). To generate enzyme chimeras, DNA sequences coding for the enzyme part of interest were amplified by PCR. PCR products were purified using the Wizard SV Gel and PCR Clean-Up System (Promega) and served as megaprimers in a site-directed mutagenesis PCR. Correct sequences were verified by sequence analysis.

### Expression and purification of recombinant enzymes

Recombinant enzymes carrying an N-terminal His_6_ tag were expressed in *E. coli* BL21 (DE3) cca::cam lacking the gene for the endogenous CCA-adding enzyme. The proteins were purified *via* affinity chromatography on a HisTrap FF (GE HealthCare) and subsequent size exclusion on a HiLoad 16/60 Superdex 75 pg column (24). Purity of enzyme preparations was monitored by SDS-PAGE electrophoresis, and protein concentration was determined by microvolume spectrophotometry. Recombinant proteins were stored at −80 °C containing 10% (v/v) glycerol until use.

### *In vitro* transcription of tRNAs

tRNA substrates were generated as T7 *in vitro* transcripts lacking the CCA-end in the presence of α−^32^P-ATP (3000 Ci/mmol). Homogenous 5′- and 3′-ends of the tRNA transcripts were generated by the cleavage reaction of flanking ribozyme cassettes (24, 51).

### Nucleotide incorporation tests and determination of arbitrary units

Nucleotide incorporation assays were performed as described (52, 53). For each experiment, at least three technical replicates were conducted. To compare enzymatic activities of different protein preparations, the efficiency of CCA addition was normalized based on the standard substrate tRNA^Phe^ from *Saccharomyces cerevisiae* (24, 48). One arbitrary unit was defined as the enzyme amount required for 50% substrate turnover.

A comparative quantitation of the individual enzymatic activities is summarized in Fig. S1. For quantitation, ImageQuant TL 8.2 software (Cytiva) was used. To this end, the band intensity for each added nucleotide in the polyacrylamide gels was quantified relative to the total signal intensities of each lane.

### Electrophoretic mobility shift assays

Binding affinities of recombinant enzymes to various tRNA substrates were determined as described (24, 48). Briefly, 0.5 pmol of α^32^P-ATP-labeled tRNA substrate were incubated with recombinant CCA-adding enzyme at a concentration of 0 to 4 μM at 20° C for 10 min. Glycerol was added to a final concentration of 18.5%. tRNAs and formed complex were separated on a 5% native polyacrylamide gel. The percentage of bound tRNA was calculated by quantifying band intensity with ImageQuant TL. Experiments were performed in three technical replicates. Mean and standard deviations were calculated using GraphPad Prism 7 (GraphPad Software, Inc). Dissociation constants were determined by nonlinear regression.

## Data availability

All presented data are contained within the article.

## Supporting information

This article contains supporting information (55, 56).

## Conflict of interest

The authors declare that they have no conflicts of interest with the contents of this article.

## References

[1] Krahn N., Fischer J.T., Söll D. (2020). Naturally occurring tRNAs with non-canonical structures. Front. Microbiol..

[2] Giegé R., Jühling F., Pütz J., Stadler P., Sauter C., Florentz C. (2012). Structure of transfer RNAs: similarity and variability. Wiley Inter. Rev. RNA.

[3] Kim S.H., Suddath F.L., Quigley G.J., McPherson A., Sussmann J.L., Wang A.H. (1974). Three-dimensional tertiary structure of yeast phenylalanine transfer RNA. Science.

[4] Sprinzl M., Cramer F. (1979). The -C-C-A end of tRNA and its role in protein biosynthesis. Prog. Nucleic Acid Res. Mol. Biol..

[5] Green R., Noller H.F. (1997). Ribosomes and translation. Annu. Rev. Biochem..

[6] Helm M., Brule H., Friede D., Giege R., Putz D., Florentz C. (2000). Search for characteristic structural features of mammalian mitochondrial tRNAs. RNA.

[7] Okimoto R., Wolstenholme D.R. (1990). A set of tRNAs that lack either the T psi C arm or the dihydrouridine arm: towards a minimal tRNA adaptor. EMBO J..

[8] Watanabe Y., Tsurui H., Ueda T., Furushima R., Takamiya S., Kita K. (1994). Primary and higher order structures of nematode (Ascaris suum) mitochondrial tRNAs lacking either the T or D stem. J. Biol. Chem..

[9] Wolstenholme, Macfarlane J.L., Okimoto R., Clary D.O., Wahleithner J.A. (1987). Bizarre tRNAs inferred from DNA sequences of mitochondrial genomes of nematode worms. Proc. Natl. Acad. Sci. U. S. A..

[10] de Bruijn M.H., Schreier P.H., Eperon I.C., Barrell B.G., Chen E.Y., Armstrong P.W. (1980). A mammalian mitochondrial serine transfer RNA lacking the "dihydrouridine" loop and stem. Nucleic Acids Res..

[11] Klimov P.B., OConnor B.M. (2009). Improved tRNA prediction in the American house dust mite reveals widespread occurrence of extremely short minimal tRNAs in acariform mites. BMC Genomics.

[12] Jühling F., Pütz J., Bernt M., Donath A., Middendorf M., Florentz C. (2012). Improved systematic tRNA gene annotation allows new insights into the evolution of mitochondrial tRNA structures and into the mechanisms of mitochondrial genome rearrangements. Nucleic Acids Res..

[13] Pons J., Bover P., Bidegaray-Batista L., Arnedo M.A. (2019). Arm-less mitochondrial tRNAs conserved for over 30 millions of years in spiders. BMC Genomics.

[14] Wende S., Platzer E.G., Jühling F., Pütz J., Florentz C., Stadler P.F. (2014). Biological evidence for the world's smallest tRNAs. Biochimie.

[15] Adrián-Serrano S., Lozano-Fernandez J., Pons J., Rozas J., Arnedo M.A. (2021). On the shoulder of giants: mitogenome recovery from non-targeted genome projects for phylogenetic inference and molecular evolution studies. J. Zool. Syst. Evol. Res..

[16] Ozerova I., Fallmann J., Mörl M., Bernt M., Prohaska S.J., Stadler P.F. (2024). Aberrant mitochondrial tRNA genes appear frequently in animal evolution. Genome Biol. Evol..

[17] Jühling F., Pütz J., Florentz C., Stadler P.F. (2012). Armless mitochondrial tRNAs in enoplea (Nematoda). Rna Biol..

[18] Jühling T., Duchardt-Ferner E., Bonin S., Wöhnert J., Pütz J., Florentz C. (2018). Small but large enough: structural properties of armless mitochondrial tRNAs from the nematode Romanomermis culicivorax. Nucleic Acids Res..

[19] Betat H., Rammelt C., Mörl M. (2010). tRNA nucleotidyltransferases: ancient catalysts with an unusual mechanism of polymerization. Cell Mol. Life Sci..

[20] Weiner A.M. (2004). tRNA maturation: RNA polymerization without a nucleic acid template. Curr. Biol..

[21] Xiong Y., Steitz T.A. (2006). A story with a good ending: tRNA 3'-end maturation by CCA-adding enzymes. Curr. Opin. Struct. Biol..

[22] Nagaike T., Suzuki T., Tomari Y., Takemoto-Hori C., Negayama F., Watanabe K. (2001). Identification and characterization of mammalian mitochondrial tRNA nucleotidyltransferases. J. Biol. Chem..

[23] Reichert A.S., Thurlow D.L., Mörl M. (2001). A eubacterial origin for the human tRNA nucleotidyltransferase?. Biol. Chem..

[24] Hennig O., Philipp S., Bonin S., Rollet K., Kolberg T., Jühling T. (2020). Adaptation of the Romanomermis culicivorax CCA-adding enzyme to miniaturized armless tRNA substrates. Int. J. Mol. Sci..

[25] Chen J.Y., Joyce P.B., Wolfe C.L., Steffen M.C., Martin N.C. (1992). Cytoplasmic and mitochondrial tRNA nucleotidyltransferase activities are derived from the same gene in the yeast Saccharomyces cerevisiae. J. Biol. Chem..

[26] Martin G., Keller W. (2007). RNA-specific ribonucleotidyl transferases. RNA (New York, N.Y.).

[27] Li F., Xiong Y., Wang J., Cho H.D., Tomita K., Weiner A.M. (2002). Crystal structures of the Bacillus stearothermophilus CCA-adding enzyme and its complexes with ATP or CTP. Cell.

[28] Tomita K., Fukai S., Ishitani R., Ueda T., Takeuchi N., Vassylyev D.G. (2004). Structural basis for template-independent RNA polymerization. Nature.

[29] Tretbar S., Neuenfeldt A., Betat H., Mörl M. (2011). An inhibitory C-terminal region dictates the specificity of A-adding enzymes. Proc. Natl. Acad. Sci. U. S. A..

[30] Yamashita S., Martinez A., Tomita K. (2015). Measurement of acceptor-TΨC helix length of tRNA for terminal A76-addition by A-adding enzyme. Structure.

[31] Okimoto R., Macfarlane J.L., Clary D.O., Wolstenholme D.R. (1992). The mitochondrial genomes of two nematodes, Caenorhabditis elegans and Ascaris suum. GENETICS.

[32] Arita M., Suematsu T., Osanai A., Inaba T., Kamiya H., Kita K. (2006). An evolutionary ‘intermediate state' of mitochondrial translation systems found in Trichinella species of parasitic nematodes: co-evolution of tRNA and EF-Tu. Nucleic Acids Res..

[33] Ohtsuki T., Watanabe Y., Takemoto C., Kawai G., Ueda T., Kita K. (2001). An "elongated" translation elongation factor Tu for truncated tRNAs in nematode mitochondria. J. Biol. Chem..

[34] Sakurai M., Watanabe Y.-I., Watanabe K., Ohtsuki T. (2006). A protein extension to shorten RNA: elongated elongation factor-Tu recognizes the D-arm of T-armless tRNAs in nematode mitochondria. Biochem. J..

[35] Watanabe Y.-I., Suematsu T., Ohtsuki T. (2014). Losing the stem-loop structure from metazoan mitochondrial tRNAs and co-evolution of interacting factors. Front. Genet..

[36] Augustin M.A., Reichert A.S., Betat H., Huber R., Mörl M., Steegborn C. (2003). Crystal structure of the human CCA-adding enzyme: insights into template-independent polymerization. J. Mol. Biol..

[37] Lizano E., Scheibe M., Rammelt C., Betat H., Mörl M. (2008). A comparative analysis of CCA-adding enzymes from human and E. coli: differences in CCA addition and tRNA 3'-end repair. Biochimie.

[38] Lusetti S.L., Wood E.A., Fleming C.D., Modica M.J., Korth J., Abbott L. (2003). C-terminal deletions of the Escherichia coli RecA protein. Characterization of *in vivo* and *in vitro* effects. J. Biol. Chem..

[39] Shi H., Moore P.B. (2000). The crystal structure of yeast phenylalanine tRNA at 1.93 A resolution: a classic structure revisited. RNA.

[40] Toh Y., Takeshita D., Numata T., Fukai S., Nureki O., Tomita K. (2009). Mechanism for the definition of elongation and termination by the class II CCA-adding enzyme. EMBO J..

[41] Deutscher M.P. (1990). Ribonucleases, tRNA nucleotidyltransferase, and the 3' processing of tRNA. Prog. Nucleic Acid Res. Mol. Biol..

[42] Salinas-Giegé T., Giegé R., Giegé P. (2015). tRNA biology in mitochondria. Int. J. Mol. Sci..

[43] Ernst F.G.M., Rickert C., Bluschke A., Betat H., Steinhoff H.-J., Mörl M. (2015). Domain movements during CCA-addition: a new function for motif C in the catalytic core of the human tRNA nucleotidyltransferases. RNA Biol..

[44] Kim S., Liu C., Halkidis K., Gamper H.B., Hou Y.-M. (2009). Distinct kinetic determinants for the stepwise CCA addition to tRNA. RNA.

[45] Kuhn C.-D., Wilusz J.E., Zheng Y., Beal P.A., Joshua-Tor L. (2015). On-enzyme refolding permits small RNA and tRNA surveillance by the CCA-adding enzyme. Cell.

[46] Ohtsuki T., Sato A., Watanabe Y.-I., Watanabe K. (2002). A unique serine-specific elongation factor Tu found in nematode mitochondria. Nat. Struct. Biol..

[47] Ohtsuki T., Watanabe Y.-I. (2007). T-armless tRNAs and elongated elongation factor Tu. IUBMB life.

[48] Hager M., Pöhler M.-T., Reinhardt F., Wellner K., Hübner J., Betat H. (2022). Substrate affinity versus catalytic efficiency: ancestral sequence reconstruction of tRNA nucleotidyltransferases solves an enzyme puzzle. Mol. Biol. Evol..

[49] Sievers F., Higgins D.G. (2021). The clustal Omega multiple alignment package. Methods Mol. Biol..

[50] Waterhouse A.M., Procter J.B., Martin D.M.A., Clamp M., Barton G.J. (2009). Jalview Version 2--a multiple sequence alignment editor and analysis workbench. Bioinformatics.

[51] Schürer H., Lang K., Schuster J., Mörl M. (2002). A universal method to produce in vitro transcripts with homogeneous 3' ends. Nucleic Acids Res..

[52] Wende S., Bonin S., Götze O., Betat H., Mörl M. (2015). The identity of the discriminator base has an impact on CCA addition. Nucleic Acids Res..

[53] Ernst F.G.M., Erber L., Sammler J., Jühling F., Betat H., Mörl M. (2018). Cold adaptation of tRNA nucleotidyltransferases: a tradeoff in activity, stability and fidelity. RNA Biol..

[54] Leibovitch M., Reid N.E., Victoria J., Hanic-Joyce P.J., Joyce P.B.M. (2019). Analysis of the pathogenic I326T variant of human tRNA nucleotidyltransferase reveals reduced catalytic activity and thermal stability in vitro linked to a conformational change. Biochim. Biophys. Acta Proteins Proteomics.

[55] Schiffer P.H., Kroiher M., Kraus C., Koutsovoulos G.D., Kumar S., Camps J.I.R. (2013). The genome of Romanomermis culicivorax: revealing fundamental changes in the core developmental genetic toolkit in Nematoda. BMC genomics.

[56] Guiglielmoni N., Villegas L.I., Kirangwa J., Schiffer P.H. (2024). Revisiting genomes of non-model species with long reads yields new insights into their biology and evolution. Front. Genet..

