# Supplementary material for: An alternative adaptation strategy of the CCA-adding enzyme to accept noncanonical tRNA substrates in Ascaris suum
Source: J Biol Chem. 2025 Mar 17;301(4):108414. doi: 10.1016/j.jbc.2025.108414 (PMC12013499; doi:10.1016/j.jbc.2025.108414)
Supplement: Supporting Information [file mmc1.docx]

**Supporting Information**


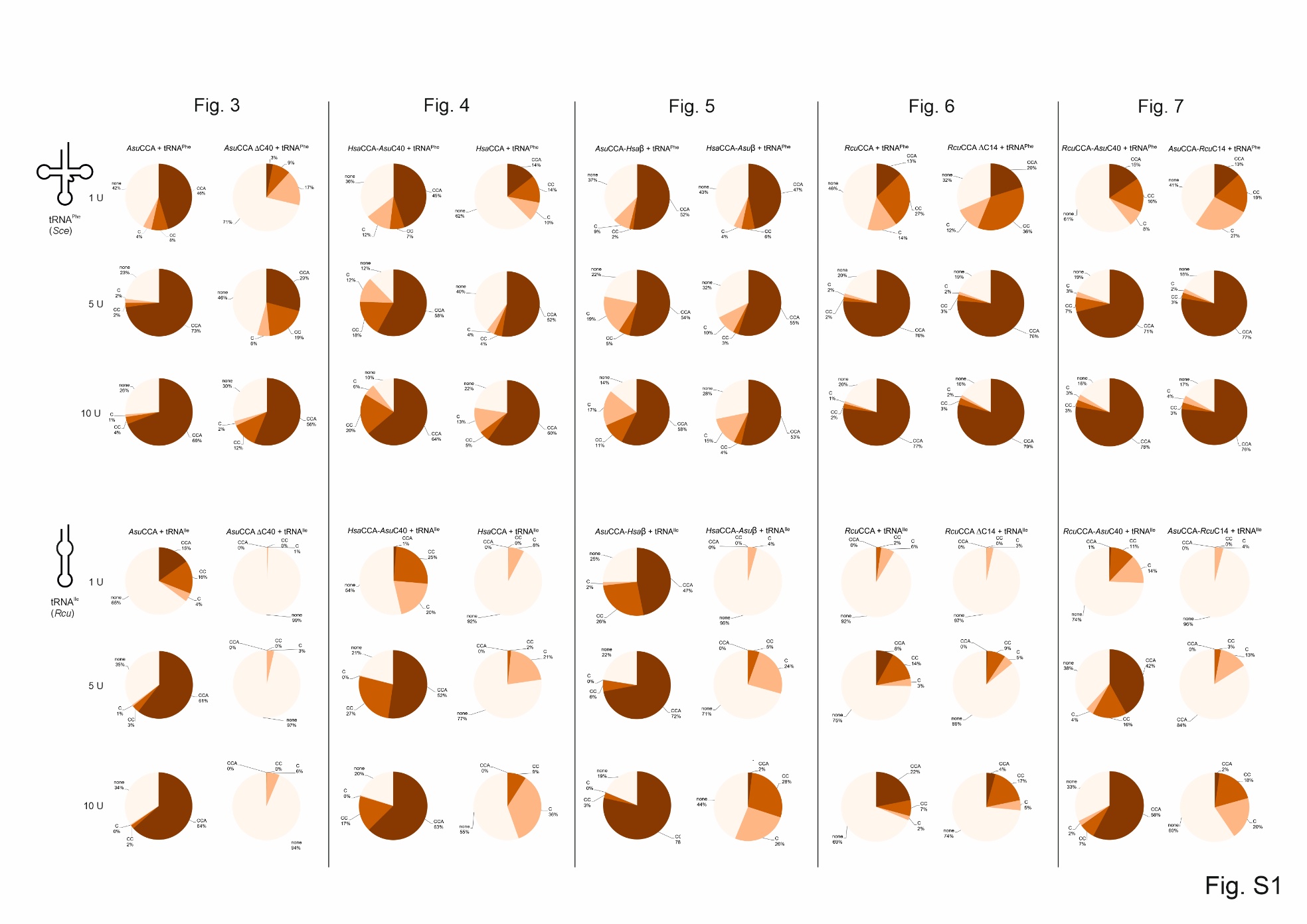


**Figure S1. Quantitation of CCA-addition.** For all tested enzyme versions, the band intensity for each added nucleotide in the polyacrylamide gels was quantified relative to the total signal intensities. The color code in the pie charts ranges from “no nucleotide addition” (light ocher) to “full CCA-addition” (dark brown).


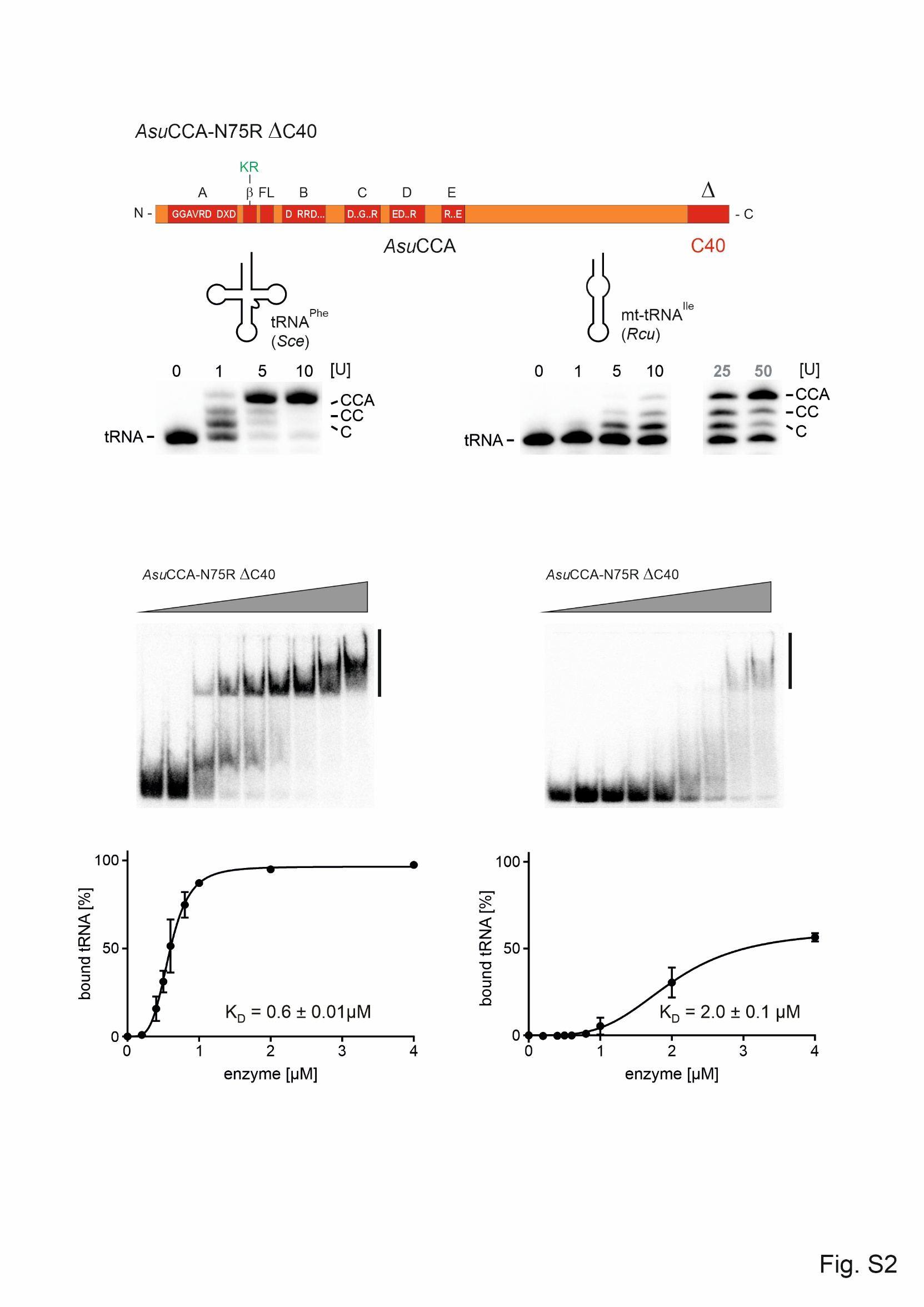


**Figure S2. Basic amino acids in the β‑turn rescue CCA-addition.** Whereas the C‑terminal deletion variant of *Asu*CCA is not able to add a complete CCA-end on the armless tRNA (see Fig. 3), the introduction of N75R replacement (resulting in the pair of basic positions KR (green)) re-establishes this activity at increased enzyme concentrations. In addition, the basic residues convey binding to this tRNA, although at a somewhat increased dissociation constant.

**
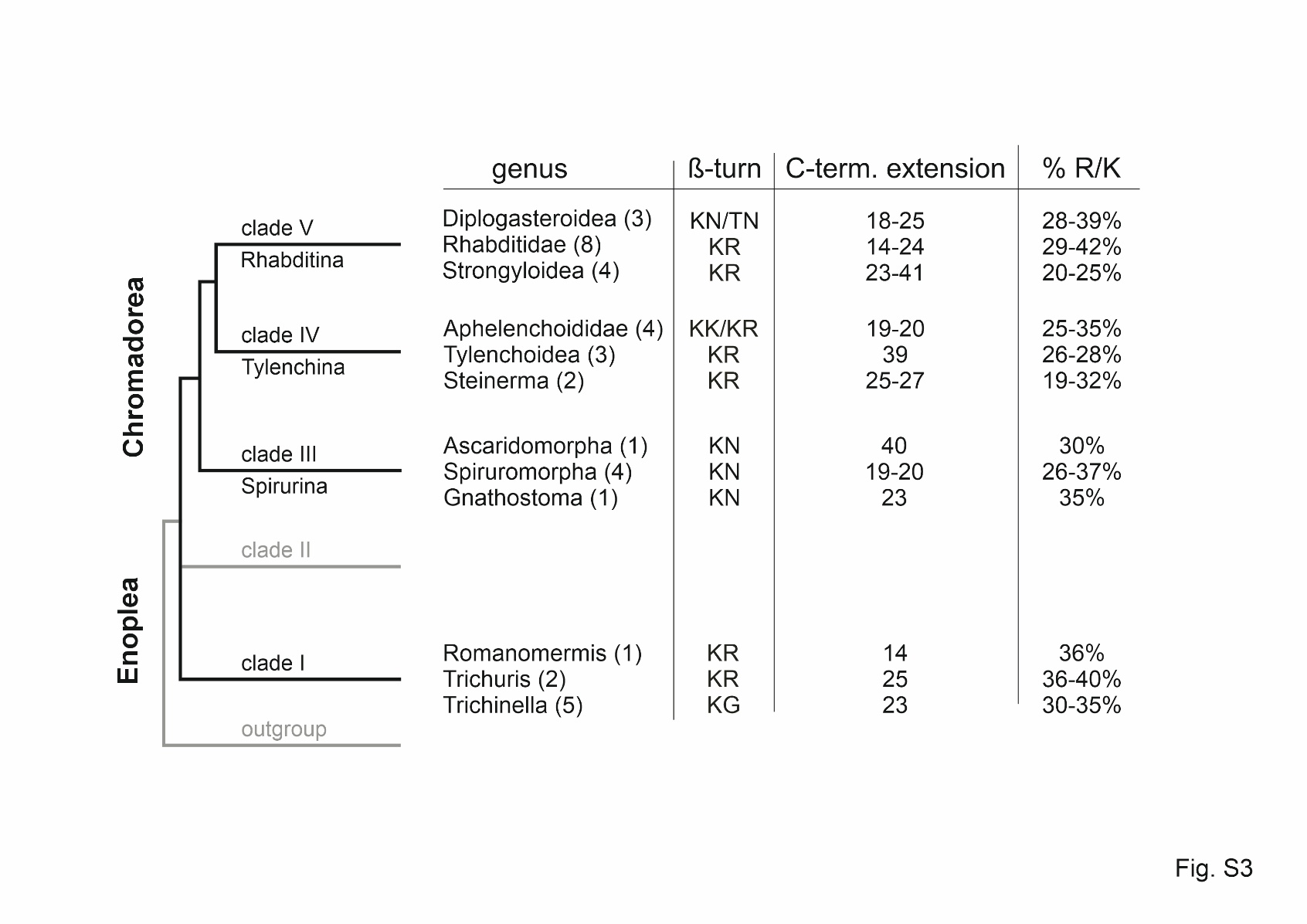
**

**Figure S3. Phylogenetic analysis of enzyme elements adapted to non-conventional tRNAs.** A nematode phylogenetic tree (1) was used to illustrate the distribution of β‑turn and C‑terminus adaptations. All clades show a C‑terminal extension (14 - 41 positions) enriched in basic amino acids (19 - 42%). In contrast, the pair of basic residues in the β‑turn element is less conserved, and no correlation between extension and adapted β‑turn is visible. Hence, individual enzymes obviously rely either on the C‑terminal extension alone or in combination with the β‑turn adaptation. In total, 38 sequences were analyzed (the various sequence numbers for each genus are indicated in brackets). For clade II (grey), no genomic data are available (1).


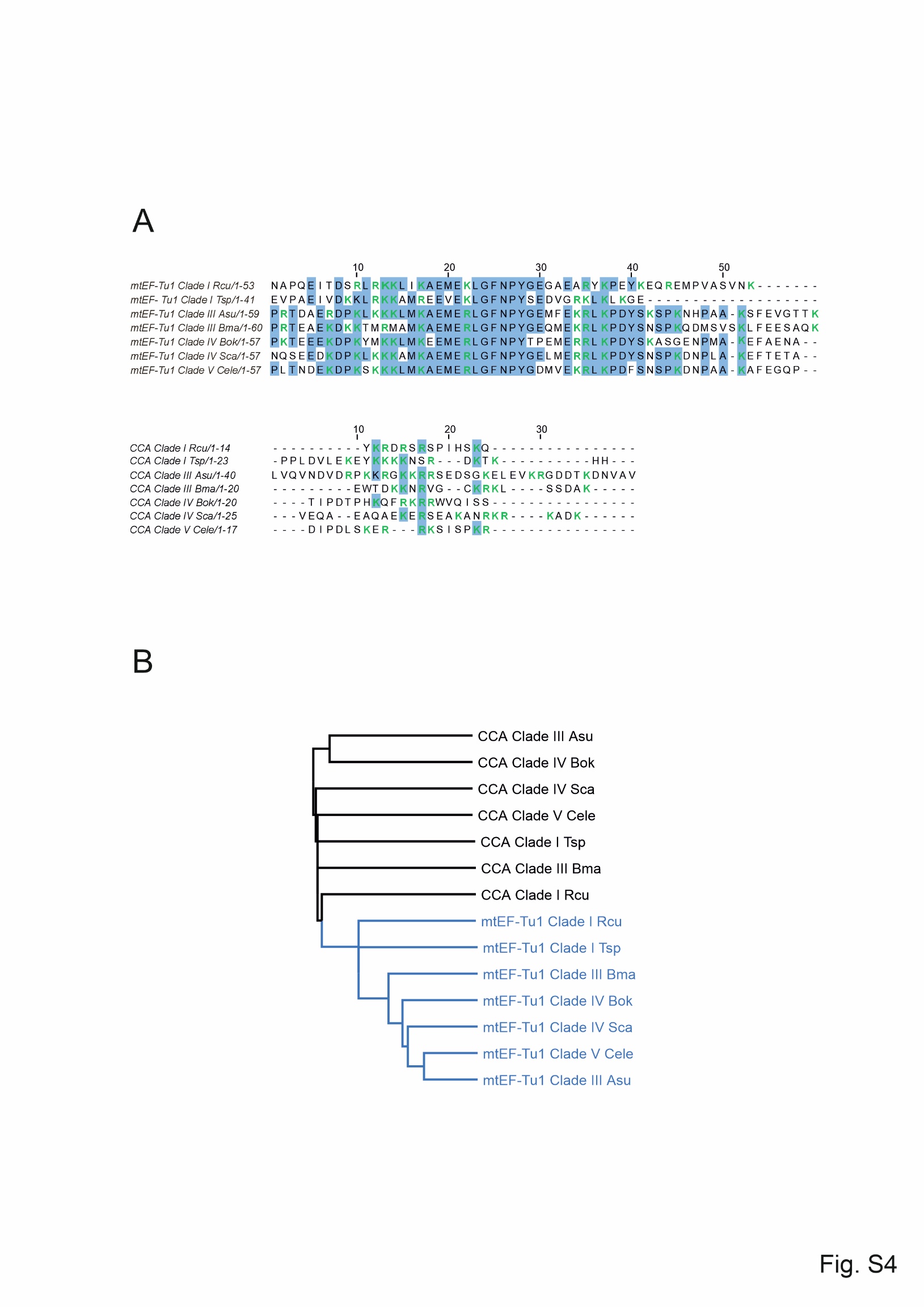


**Figure S4. C‑terminal extensions in mitochondrial EF‑Tu1 and CCA-adding enzymes of representative organisms from different nematode clades.** **(A)** Alignment of C‑terminal extensions. While the mt EF‑Tu1 proteins exhibit a high sequence conservation, the extensions of CCA-adding enzymes are highly divergent. Yet, both types of extensions are enriched in basic residues (green) that probably contribute to the binding of armless tRNAs. **(B)** A phylogenetic analysis of the C‑terminal extensions indicates a common evolutionary origin for EF‑Tu1 sequences (blue), while the extension of CCA-adding enzymes do not show such a close relationship (black).

*Asu*: *Ascaris suum* (CCA: UniProt F1L3Q5; EFTu1: BAF31894.1)

*Bma*: *Brugia malayi* (CCA: CRZ24724.1; EFTu1: XP_001902322.1)

*Bok*: *Bursaphelenchus okinawaensis* (CCA: CAD5224554.1; EFTu1: CAD5225554.1)

*Cele*: *Caenorhabditis elegans* (CCA: NP_001023976.1; EFTu1: NP_497623.1)

*Rcu*: *Romanomermis culicivorax* (CCA; EFTu1: ENA PRJEB66727 (2))

*Sca*: *Steinernema carpocapsae* (CCA: TKR86463; EFTu1: TKR75678.1)

*Tsp*: *Trichinella spiralis (*CCA: KAL1230143.1; EFTu1: KRY39081.1)

References

1. Schiffer, P. H., Kroiher, M., Kraus, C., Koutsovoulos, G. D., Kumar, S., Camps, J. I. R., Nsah, N. A., Stappert, D., Morris, K., Heger, P., Altmüller, J., Frommolt, P., Nürnberg, P., Thomas, W. K., Blaxter, M. L., and Schierenberg, E. (2013) The genome of Romanomermis culicivorax: revealing fundamental changes in the core developmental genetic toolkit in Nematoda. *BMC genomics* **14**, 923 10.1186/1471-2164-14-923 PMID 24373391

2. Guiglielmoni, N., Villegas, L. I., Kirangwa, J., and Schiffer, P. H. (2024) Revisiting genomes of non-model species with long reads yields new insights into their biology and evolution. *Front. Genet.* **15**, 1308527 10.3389/fgene.2024.1308527 PMID 38384712

**Enzyme Sequences**

**CCA-adding enzymes, wt**

*Asu*CCA

MKLDSEEFRSLFTPELKKLNDLFIANKFQLRMAGGAVRDLLMGLRPADIDFASDATPSQMKELFTREGIRMLNKNGEEHGTITCRIDDKENFEITTLRIDVVCDGRRAKVEFTTDWQLDANRRDLTINSLFLELDGTVIDYFGGIEDLRARRVAFVGDATQRIQEDYLRILRYFRFFGRIAKSGDAHEQTTLDAIINNKQGLMNISGERIWSELKKICVGRFGGDVLTTMVSRCGLASLLGLPEDCDVATCREIFNYNSDRSLEPMTVLSALFKNQQDIDVFHKRCKLSNAEKSLAEFIVEKRDEARSNIDSTKYFKDLILDYEKAPGRDKKKINGRDMVRELAKYVCASAEIIEEIGVWKMPEFPVSGKDLIGTGVKTGPIMRRVLTHLFELWKKSEYKAERDELLSHITPELVQVNDVDRPKKRGKKRRSEDSGKELEVKRGDDTKDNVAV

*Hsa*CCA

MKLQSPEFQSLFTEGLKSLTELFVKENHELRIAGGAVRDLLNGVKPQDIDFATTATPTQMKEMFQSAGIRMINNRGEKHGTITARLHEENFEITTLRIDVTTDGRHAEVEFTTDWQKDAERRDLTINSMFLGFDGTLFDYFNGYEDLKNKKVRFVGHAKQRIQEDYLRILRYFRFYGRIVDKPGDHDPETLEAIAENAKGLAGISGERIWVELKKILVGNHVNHLIHLIYDLDVAPYIGLPANASLEEFDKVSKNVDGFSPKPVTLLASLFKVQDDVTKLDLRLKIAKEEKNLGLFIVKNRKDLIKATDSSDPLKPYQDFIIDSREPDATTRVCELLKYQGEHCLLKEMQQWSIPPFPVSGHDIRKVGISSGKEIGALLQQLREQWKKSGYQMEKDELLSYIKKT

*Rcu*CCA

MKIDSPQFRSIFTPELRIVSDLFEKNGFELRIAGGAVRDILLGQVPHDIDFATTATPTQMINLFNKEGIRMLNKRGEKHGTITCRINEKVNFEITTLRIDKLCDGRRAEVEFTTDWYKDANRRDLTVNSIFLGLDGTVYDYFDGIEHLKARKILFVGDAKTRIQEDYLRILRYFRFFGRLTITPDDHDPMTLLAIKDNVHGLKNVSGERLWTEFKRIVTGRFAGSVVKIMLECNIGSYLGLPETCNIDEFIRLCDVGCLAHNPMSMTMVSALLNFEDDIYNLDGRLKLSNKERFLGNFIIEHRSKISRKKSKDDSVESYYKDLLIGFQSPSIKLENSKEYVLELAKYNGDFDISRILEDYQAPKFPVDGVMLLQWGVKKGPAMKAVLSKLFEFWKIKQYDVTNDEFFQQIDDCYKRDRSRSPIHSKQ

**CCA-adding enzymes, deletion variants**

*Asu*CCA ΔC40

MKLDSEEFRSLFTPELKKLNDLFIANKFQLRMAGGAVRDLLMGLRPADIDFASDATPSQMKELFTREGIRMLNKNGEEHGTITCRIDDKENFEITTLRIDVVCDGRRAKVEFTTDWQLDANRRDLTINSLFLELDGTVIDYFGGIEDLRARRVAFVGDATQRIQEDYLRILRYFRFFGRIAKSGDAHEQTTLDAIINNKQGLMNISGERIWSELKKICVGRFGGDVLTTMVSRCGLASLLGLPEDCDVATCREIFNYNSDRSLEPMTVLSALFKNQQDIDVFHKRCKLSNAEKSLAEFIVEKRDEARSNIDSTKYFKDLILDYEKAPGRDKKKINGRDMVRELAKYVCASAEIIEEIGVWKMPEFPVSGKDLIGTGVKTGPIMRRVLTHLFELWKKSEYKAERDELLSHITPE

*Rcu*CCA ΔC14

MKIDSPQFRSIFTPELRIVSDLFEKNGFELRIAGGAVRDILLGQVPHDIDFATTATPTQMINLFNKEGIRMLNKRGEKHGTITCRINEKVNFEITTLRIDKLCDGRRAEVEFTTDWYKDANRRDLTVNSIFLGLDGTVYDYFDGIEHLKARKILFVGDAKTRIQEDYLRILRYFRFFGRLTITPDDHDPMTLLAIKDNVHGLKNVSGERLWTEFKRIVTGRFAGSVVKIMLECNIGSYLGLPETCNIDEFIRLCDVGCLAHNPMSMTMVSALLNFEDDIYNLDGRLKLSNKERFLGNFIIEHRSKISRKKSKDDSVESYYKDLLIGFQSPSIKLENSKEYVLELAKYNGDFDISRILEDYQAPKFPVDGVMLLQWGVKKGPAMKAVLSKLFEFWKIKQYDVTNDEFFQQIDDC

**CCA-adding enzymes, chimeric forms**

Introduced sequences are indicated in red

*Hsa*CCA-*Asu*C40

MKLQSPEFQSLFTEGLKSLTELFVKENHELRIAGGAVRDLLNGVKPQDIDFATTATPTQMKEMFQSAGIRMINNRGEKHGTITARLHEENFEITTLRIDVTTDGRHAEVEFTTDWQKDAERRDLTINSMFLGFDGTLFDYFNGYEDLKNKKVRFVGHAKQRIQEDYLRILRYFRFYGRIVDKPGDHDPETLEAIAENAKGLAGISGERIWVELKKILVGNHVNHLIHLIYDLDVAPYIGLPANASLEEFDKVSKNVDGFSPKPVTLLASLFKVQDDVTKLDLRLKIAKEEKNLGLFIVKNRKDLIKATDSSDPLKPYQDFIIDSREPDATTRVCELLKYQGEHCLLKEMQQWSIPPFPVSGHDIRKVGISSGKEIGALLQQLREQWKKSGYQMEKDELLSYIKKTLVQVNDVDRPKKRGKKRRSEDSGKELEVKRGDDTKDNVAV

*Asu*CCA-*Rcu*C14

MKLDSEEFRSLFTPELKKLNDLFIANKFQLRMAGGAVRDLLMGLRPADIDFASDATPSQMKELFTREGIRMLNKNGEEHGTITCRIDDKENFEITTLRIDVVCDGRRAKVEFTTDWQLDANRRDLTINSLFLELDGTVIDYFGGIEDLRARRVAFVGDATQRIQEDYLRILRYFRFFGRIAKSGDAHEQTTLDAIINNKQGLMNISGERIWSELKKICVGRFGGDVLTTMVSRCGLASLLGLPEDCDVATCREIFNYNSDRSLEPMTVLSALFKNQQDIDVFHKRCKLSNAEKSLAEFIVEKRDEARSNIDSTKYFKDLILDYEKAPGRDKKKINGRDMVRELAKYVCASAEIIEEIGVWKMPEFPVSGKDLIGTGVKTGPIMRRVLTHLFELWKKSEYKAERDELLSHITPEYKRDRSRSPIHSKQ

*Rcu*CCA-*Asu*C40

MKIDSPQFRSIFTPELRIVSDLFEKNGFELRIAGGAVRDILLGQVPHDIDFATTATPTQMINLFNKEGIRMLNKRGEKHGTITCRINEKVNFEITTLRIDKLCDGRRAEVEFTTDWYKDANRRDLTVNSIFLGLDGTVYDYFDGIEHLKARKILFVGDAKTRIQEDYLRILRYFRFFGRLTITPDDHDPMTLLAIKDNVHGLKNVSGERLWTEFKRIVTGRFAGSVVKIMLECNIGSYLGLPETCNIDEFIRLCDVGCLAHNPMSMTMVSALLNFEDDIYNLDGRLKLSNKERFLGNFIIEHRSKISRKKSKDDSVESYYKDLLIGFQSPSIKLENSKEYVLELAKYNGDFDISRILEDYQAPKFPVDGVMLLQWGVKKGPAMKAVLSKLFEFWKIKQYDVTNDEFFQQIDDCLVQVNDVDRPKKRGKKRRSEDSGKELEVKRGDDTKDNVAV

*Asu*CCA-*Hsa*β

MKLDSEEFRSLFTPELKKLNDLFIANKFQLRMAGGAVRDLLMGLRPADIDFASDATPSQMKEMFQSAGIRMINNRGEKHGTITARLHEENFEITTLRIDVVCDGRRAKVEFTTDWQLDANRRDLTINSLFLELDGTVIDYFGGIEDLRARRVAFVGDATQRIQEDYLRILRYFRFFGRIAKSGDAHEQTTLDAIINNKQGLMNISGERIWSELKKICVGRFGGDVLTTMVSRCGLASLLGLPEDCDVATCREIFNYNSDRSLEPMTVLSALFKNQQDIDVFHKRCKLSNAEKSLAEFIVEKRDEARSNIDSTKYFKDLILDYEKAPGRDKKKINGRDMVRELAKYVCASAEIIEEIGVWKMPEFPVSGKDLIGTGVKTGPIMRRVLTHLFELWKKSEYKAERDELLSHITPELVQVNDVDRPKKRGKKRRSEDSGKELEVKRGDDTKDNVAV

*Hsa*CCA-*Asu*β

MKLQSPEFQSLFTEGLKSLTELFVKENHELRIAGGAVRDLLNGVKPQDIDFATTATPTQMKELFTREGIRMLNKNGEEHGTITCRIDDKENFEITTLRIDVTTDGRHAEVEFTTDWQKDAERRDLTINSMFLGFDGTLFDYFNGYEDLKNKKVRFVGHAKQRIQEDYLRILRYFRFYGRIVDKPGDHDPETLEAIAENAKGLAGISGERIWVELKKILVGNHVNHLIHLIYDLDVAPYIGLPANASLEEFDKVSKNVDGFSPKPVTLLASLFKVQDDVTKLDLRLKIAKEEKNLGLFIVKNRKDLIKATDSSDPLKPYQDFIIDSREPDATTRVCELLKYQGEHCLLKEMQQWSIPPFPVSGHDIRKVGISSGKEIGALLQQLREQWKKSGYQMEKDELLSYIKKT

*Asu*CCA ΔC40 N75R

MKLDSEEFRSLFTPELKKLNDLFIANKFQLRMAGGAVRDLLMGLRPADIDFASDATPSQMKELFTREGIRMLNKRGEEHGTITCRIDDKENFEITTLRIDVVCDGRRAKVEFTTDWQLDANRRDLTINSLFLELDGTVIDYFGGIEDLRARRVAFVGDATQRIQEDYLRILRYFRFFGRIAKSGDAHEQTTLDAIINNKQGLMNISGERIWSELKKICVGRFGGDVLTTMVSRCGLASLLGLPEDCDVATCREIFNYNSDRSLEPMTVLSALFKNQQDIDVFHKRCKLSNAEKSLAEFIVEKRDEARSNIDSTKYFKDLILDYEKAPGRDKKKINGRDMVRELAKYVCASAEIIEEIGVWKMPEFPVSGKDLIGTGVKTGPIMRRVLTHLFELWKKSEYKAERDELLSHITPE
